# Are *Drosophila* preferences for yeasts stable or contextual?

**DOI:** 10.1002/ece3.5366

**Published:** 2019-06-30

**Authors:** Catrin S. Günther, Sarah J. Knight, Rory Jones, Matthew R. Goddard

**Affiliations:** ^1^ Joseph Banks Laboratories, School of Life Sciences University of Lincoln Lincoln UK; ^2^ School of Biological Sciences The University of Auckland Auckland New Zealand

**Keywords:** chemical communication, *Drosophila*, fruit, mutualism, *Saccharomycetaceae*, yeast

## Abstract

Whether there are general mechanisms, driving interspecific chemical communication is uncertain. *Saccharomycetaceae* yeast and *Drosophila* fruit flies, both extensively studied research models, share the same fruit habitat, and it has been suggested their interaction comprises a facultative mutualism that is instigated and maintained by yeast volatiles. Using choice tests, experimental evolution, and volatile analyses, we investigate the maintenance of this relationship and reveal little consistency between behavioral responses of two isolates of sympatric *Drosophila* species. While *D. melanogaster* was attracted to a range of different *Saccharomycetaceae* yeasts and this was independent of fruit type, *D. simulans* preference appeared specific to a particular *S. cerevisiae* genotype isolated from a vineyard fly population. This response, however, was not consistent across fruit types and is therefore context‐dependent. In addition, *D. simulans* attraction to an individual *S. cerevisiae* isolate was pliable over ecological timescales. Volatile candidates were analyzed to identify a common signal for yeast attraction, and while *D. melanogaster* generally responded to fermentation profiles, *D. simulans* preference was more discerning and likely threshold‐dependent. Overall, there is no strong evidence to support the idea of bespoke interactions with specific yeasts for either of these *Drosophila* genotypes. Rather the data support the idea *Drosophila* are generally adapted to sense and locate fruits infested by a range of fungal microbes and/or that yeast–*Drosophila* interactions may evolve rapidly.

## 1 INTRODUCTION

Chemical communication between organisms is ancient and regulates a variety of important intraspecific (Leonhardt, Menzel, Nehring, & Schmitt, 2016; Venuleo, Raven, & Giordano, 2017) and interspecific biological interactions within ecological networks (Archie & Theis, 2011; Pickett & Khan, 2016). Behavioral responses to olfactory stimuli can be both learned and intrinsic (Bergström, 2008), and natural selection may operate on traits that are heritable and correlate with fitness for both signal sender and receiver (West, Griffin, & Gardner, 2007). Pheromones are a classic example of intraspecific chemical signals that communicate social behavior beneficial to members of the same species (Leonhardt et al., 2016; Yew & Chung, 2017). Other volatile compounds serve to repulse predators (Deletre et al., 2016) and thus elevate fitness by decreasing interspecies interactions. Finally, some volatile blends, such as floral scents, may act as interspecies attractants where both the sender and receiver mutually benefit from the chemically mediated information (Raguso, 2008; Schiestl, 2010). It has been suggested that chemical signals evolve from unintentional cues (Steiger, Schmitt, & Schaefer, 2011; Weiss et al., 2013), but these are often defined by complex blends of volatiles rather than single key compounds, and the volatiles implicated might have multiple biological functions (Kessler, Diezel, Clark, Colquhoun, & Baldwin, 2013; Tan & Nishida, 2012). However, once chemically mediated facultative mutualistic interactions between species are established, it is not clear how robust they are nor how they evolve (Buser, Newcomb, Gaskett, & Goddard, 2014). This means we are unable to predict whether all members of a species show the same extent of behavioral response to the same chemical cues and thus have no understanding of how stable or fluctuating these interactions are in nature.

As it stands, we are unable to predict the extent of variance in the efficacy of communication and thus interaction between members of facultative interacting species: Are there differences in chemosensory preference or perception within species? How similarly do closely related species sense and respond equally to mutualistic partners? Overall, we have no understanding of how stable or changeable chemically mediated facultative mutualistic interactions are in nature.

The role of fungal volatiles as semiochemicals attracting insects is well described (Beck & Vannette, 2017; Madden et al., 2018) and *Saccharomycetaceae* (budding) yeasts and *Drosophila* flies in the “melanogaster” subgroup (Clark et al., 2007) are not only influential research models but also co‐inhabit economically important fruit crops (Hamby, Hernandez, Boundy‐Mills, & Zalom, 2012; Lam & Howell, 2015) where certain *Drosophila* species (such as *D. suzukii*) may act as nuisance and damaging pests (Walsh et al., 2011), and yeasts may variously have negative, benign or positive impacts on fruits or their fermented products (Gschaedler, 2017; Suh, Blackwell, Kurtzman, & Lachance, 2006). While a variety of *Saccharomycetaceae* yeast species are found associated with fruits (Masneuf‐Pomarede, Bely, Marullo, & Albertin, 2016; Taylor, Tsai, Anfang, Ross, & Goddard, 2014), they are also found in a range of other niches (Gayevskiy & Goddard, 2016; Morrison‐Whittle, Lee, & Goddard, 2017). There are approximately twenty genera in the *Saccharomycetaceae* family, and most tend to be associated with the early fermentation of fruits (Masneuf‐Pomarede et al., 2016; Suh et al., 2006) and *Saccharomyces cerevisiae* and *S. uvarum* tend to dominate from mid‐ferment on (Marsit & Dequin, 2015). The available data show these yeast species display significant genetic and geographic diversity (Gayevskiy & Goddard, 2016). There are well over 1,600 *Drosophila* species, which also have large genetic and geographic diversity (O'Grady & DeSalle, 2018), but of these, *Drosophila melanogaster* is the most studied. At least *D. melanogaster* olfactorial pathways appear tuned to microbial volatiles (Mansourian & Stensmyr, 2015), and these volatiles influence behavioral decisions for substrates selected for food and oviposition (Becher et al., 2012; Stökl et al., 2010).

While *Drosophila* in the *melanogaster* subgroup breed in fruit, they derive an array of fitness benefits from consuming yeasts which include influences on sexual receptivity (Gorter et al., 2016), fecundity and larvae development (Buser et al., 2014; Rohlfs & Kürschner, 2010) and other life history traits (Anagnostou, Dorsch, & Rohlfs, 2010). It is therefore unsurprising that at least *D. melanogaster* and *Drosophila simulans* are strongly attracted to certain yeast‐derived volatiles (Becher et al., 2012; Buser et al., 2014; Günther, Goddard, Newcomb, & Buser, 2015; Madden et al., 2018; Stökl et al., 2010). Yeasts metabolize fruit precursors to produce energy and biomass, but also release a range of yeast volatile organic compounds (YVOCs) as they do so (Cordente, Curtin, Varela, & Pretorius, 2012; Hazelwood, Daran, Maris, Pronk, & Dickinson, 2008). Yeasts are immotile and thus doomed to local extinction along with ephemeral fruits they inhabit. Logically, traits which increase the propensity of at least some members of a yeast colony to be transported to new habitats, which they may then colonize, will be under positive selection (Christiaens et al., 2014; Madden et al., 2018). Following this hypothesis, one *Saccharomycetaceae cerevisiae* isolate (ScNZ) has been shown to derive fitness benefits from interacting with an isofemale *D. simulans* population, indicating this interaction might comprise a mutualism (Buser et al., 2014). However, there is evidence that other *S. cerevisiae* isolates, as well as isolates from other *Saccharomycetaceae* species, are repulsive to some *Drosophila* (Buser et al., 2014; Palanca, Gaskett, Günther, Newcomb, & Goddard, 2013)*,* and so it is not yet clear how general or specific any mutualism might be (Günther & Goddard, 2019). Yeasts produce ethanol which has been shown to induce interference competition with microbes (Goddard, 2008), but the function of most YVOCs, if indeed they have any other than representing stochastic metabolic endpoints, is not at all well understood (Saerens, Delvaux, Verstrepen, & Thevelein, 2010). Here, we use the fungi–fruit–fly system as a model to test how robust facultative chemically mediated interspecies interactions are. Using preference testing, experimental evolution and volatile analysis we ask.

### 1.1 Does yeast preference differ between *Drosophila* genotypes?

*Drosophila simulans* and *D. melanogaster* are closely related (Clark et al., 2007; O'Grady & DeSalle, 2018) sympatric (Capy & Gibert, 2004) and attracted to banana and commercial (Vector 960) traps and to the yeast‐like chemical mimicry of the Solomon's lily (Stökl et al., 2010), suggesting similar chemosensory preferences in both species. However, we have previously shown that isolates from *D. simulans* and *D. melanogaster* are variably attracted to *S. cerevisiae* yeast genotypes grown in grape juice (Günther et al., 2015; Palanca et al., 2013). Whether there are specific yeast–fly pairings in which *Saccharomycetaceae* yeasts are consistently attractive to *Drosophila* is not clear.

### 1.2 Does the fruit context modulate yeast preference?

*Drosophila* attraction appears contingent on a blend of YVOCs and fruit‐derived compounds (Cordente et al., 2012) suggesting any yeast–fly associations should be considered as part of a tripartite relationship including fruits/plants. However, the impact of the fruit component on the putative yeast‐fly association has received little attention. Studies testing host plant specificity of cactophilic *D. mojavensis* show that host plant preference can shift in response to plant–microbe and also microbe–microbe interactions (Date, Crowley‐Gall, Diefendorf, & Rollmann, 2017). However, it is not known whether different fruit substrates alter the mode of any yeast‐fly interaction, and thus the degree to which the past and future evolution of yeast–fly interactions are affected by the plant host.

### 1.3 Are yeast preferences plastic or conserved?

Virtually nothing is known about the capacity for selection to operate on and change facultative chemically mediated interactions, though at least two studies show within yeast species variance for fly attraction (Buser et al., 2014; Palanca et al., 2013), suggesting attraction may potentially evolve quickly by acting on standing variance. The magnitude of potential selective effects on fly attraction will determine the likely stability of these interactions through time.

### 1.4 Are there conserved chemical mechanisms underlying *Drosophila* attraction to yeast?

Plants provide precursors in the form of sugars and amino acids for YVOCs formation. In addition to conversion of glucose to ethanol, a range of other YVOCs are produced, for example, fusel‐alcohols, such as 3‐methyl butanol (Hazelwood et al., 2008), and their corresponding acetate esters (i.e., ethyl acetate and 3‐methylbutyl acetate) have been suggested to mediate fruit fly attraction (Christiaens et al., 2014; Günther et al., 2015), and a core set of YVOCS has been proposed to act as key compounds for *D. melanogaster* attraction (Becher et al., 2012). However, the chemical preference of *D. simulans* was not driven simply by the presence or absence of these compounds but involved more subtle combinations of relative ratios of YVOCs in combination with a suite of fruit‐derived background odors (Günther et al., 2015). Single lines of both, *D. simulans* and *D. melanogaster*, were previously described as mutualistic partners for chemically mediated dispersal of *S. cerevisiae* (Buser et al., 2014; Christiaens et al., 2014). It is however not clear whether *Drosophila* attraction is mediated by a universal YVOCs signal or whether chemically mediated yeast preference is plastic and contextual and thus might arise by chance (Günther & Goddard, 2019).

## 2 METHODS

### 2.1 Fruit juice ferments

Fresh fruit was obtained from fresh‐produce markets and local farms around Lincoln (Galley Hill farm, UK), rinsed with sterile water, and juiced with a sterilized (Distel Laboratory Surface Disinfectant) kitchen juicer (Braun J‐500). Clarified juice was sterilized with dimethyl dicarbonate (1:2500; Sigma‐Aldrich) and stored at −80°C. Juice sterility was confirmed by spread‐plating on YPDA (1% yeast extract, 2% peptone, 2% dextrose, 2% agar) and Nutrient agar (Fisher bioreagents) and 5 days at 28°C and 35°C, respectively. Replicate (*n* = 6) samples of yeast isolates were inoculated into 5 ml of juice at 5 × 10^6^ cells per mL and incubated for 48h at 28°C and 200 rpm. To test for variation in attraction to different yeasts, eleven isolates consisting of eight different fruit or insect associated genera were inoculated in strawberry juice (Table SMM in Appendix S1). In an earlier study, *S. cerevisiae* strain FlyKR_78.3 (ScNZ) was isolated from *D. simulans* and found to be attractive when inoculated in Sauvignon Blanc grape juice (Buser et al., 2014; Günther et al., 2015), whereas *S. cerevisiae* DBVP6044 (ScWA; Liti et al., 2009) was shown to be repulsive. To test for the impact of the fruit context on yeast preference, attractiveness of these two *S. cerevisiae* isolates was compared when inoculated in plum (var. “Victoria”), apple (var. “Jonagold”) and strawberry juice. Total soluble solids were measured with a PAL‐1 refractometer (ATAGO™) before and after incubation to evaluate sugar consumption as proxy for fermentation progress. The supernatant was frozen at −80°C for volatile profiling and behavioral assays.

### 2.2 Behavioral study

We used an isofemale *Drosophila simulans* line derived from a vineyard population near Auckland, New Zealand (Buser et al., 2014), and the standard *D. melanogaster* Oregon R wild‐type (Carolina^®^). Both fly species were propagated at 25°C and 12:12 light:dark cycle as described earlier (Günther et al., 2015). Starved (25 hr) females (*n* = 80; 3–7 days old) were used in two‐way T‐maze choice tests (30 min in dark, 6 hr before dark cycle, *n* = 6) with 10 ml (1:1000 dilution) of each ferment and sterile juice as control (Buser et al., 2014; Günther et al., 2015; Palanca et al., 2013). Head‐to‐head competition experiments between ScNZ and ScWA were also performed in each fruit type in order to compare against preference to sterile juice. Flies were anaesthetized on ice for 5‐min before entering the T‐maze and euthanized after the experiment at −20°C. An attraction index (AI) was calculated (Buser et al., 2014) using the proportion of flies found in either arm of the T‐maze, and the binominal distribution was used to test whether the dispersal of flies between both arms of the T‐maze was significantly different from random.

### 2.3 *Drosophila* experimental evolution

**Figure 1:**
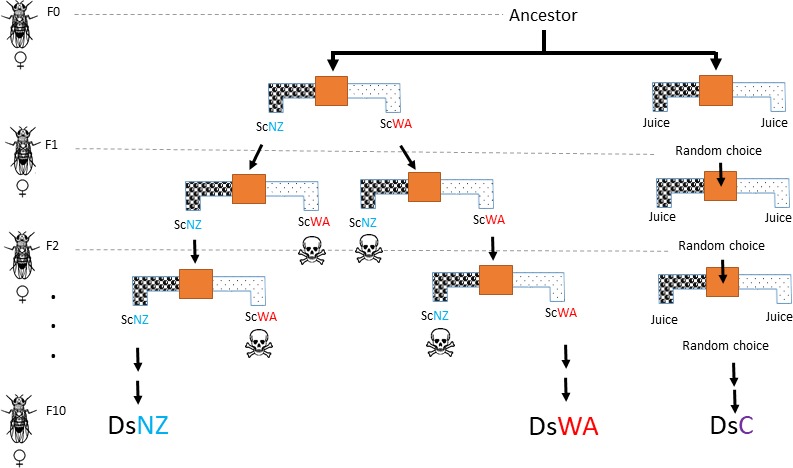
Mechanism of selection for female *Drosophila simulans* flies (Ds) with preference to an initially attractive *Saccharomyces cerevisiae* strain (ScNZ) and initially unattractive strain (ScWA) when inoculated in strawberry juice. Triplicate populations were founded for each treatment from the ancestral population and were evolved over a six month period equating to ten *Drosophila* generations. The treatments were: 1) control populations (DsC), where no selection for fly choice was applied: the next generation was founded from flies selected from one side of the T‐maze at random;  2) selection for flies choosing the yeast strain that was attractive to the founding ancestral fly population (ScNZ) and these are labeled DsNZ; and 3) selection for flies choosing the yeast strain (ScWA) that was significantly less preferred by the founding ancestral fly population, and these populations are labeled DsWA. Selection for fly preference was applied each generation by head‐to‐head competition between ScNZ and ScWA with 60–80 female flies per replicate population in duplicate. According to choice, 32–70 flies per population were transferred to fresh media for oviposition and killed after three days

The ancestral *D. simulans* population derived from a single female that was isolated from a wild population (see above) and propagated in the laboratory for 3 years using the conditions described above before the experiment. Nine populations were founded by approximately 20 females each from the base population and were evolved over a six‐month period equating to ten *Drosophila* generations per treatment (Figure 1): (a) control populations, where no selection for fly choice was applied; (b) selection for flies choosing a yeast strain that was attractive to the founding ancestral fly population in strawberry (ScNZ, AI: 0.12; *p* = 0.02); and (c) selection for flies choosing a yeast strain that was significantly less preferred by the founding ancestral fly population (ScWA, AI: −0.05, *p* = 0.01 against ScNZ in strawberry). Each treatment comprised triplicate populations. Selection for fly preference was applied in duplicate per population (*N* = 6 per treatment) and generation with 60–80 female flies per replicate. All flies (*N* = 30–60) that made the appropriate choice in head‐to‐head choice tests between ScNZ and ScWA were used to found the next generation. Control lines were subject to the same head‐to‐head choices except with sterile juice in both arms and where choice towards one side was chosen randomly to found the next generation. The first emerging progeny (3–7 days old) of each generation was used for selection and consecutive propagation as described. According to choice, female flies were immediately transferred to fresh media for oviposition and killed after three days. Selection was conducted in the same batch of strawberry juice, and yeasts were not allowed to co‐evolve with flies. The eleventh generation of flies from each population was exposed to head‐to‐head choice tests using the ancestrally attractive and unattractive yeasts (*N* = 6 per population/ *N* = 18 per treatment) and AIs were calculated. Change in fly preference (AI) during experimental evolution was evaluated by analysis of variance (ANOVA) and Tukey‐Kramer Honestly Significantly Difference (Tukey HSD) corrected post hoc tests.

### 2.4 Quantitative HS‐SPME GC‐MS

Volatile profiles were analyzed from fruit juice and cell‐free ferments using static Headspace Solidphase‐Microextraction (HS‐SPME) and Gas Chromatography–Mass Spectrometry (GC‐MS). The diluted and salt‐saturated (1.5g NaCl) sample (4 ml) was mixed with 10 uL 3‐heptanol (5 mg/L dissolved in water:ethanol 1:1) as internal standard in a 10 ml headspace vial with PTFE seal. Volatiles were extracted from equilibrated samples (30 min, 40°C) for 20 min at 40°C without agitation using a 50/30 μm DVB‐Car‐PDMS coated fiber (Supelco, 57348‐U). Compounds were separated via nonpolar GC using a 5‐MS column and desorbed thermally using a linear GC‐program (40°C hold for 2 min, then 5°C/min to 200°C and 30°C/min to 300°C) and fragmented in a Quadrupole‐MS. Total Ion Chromatogram peaks were selected when: (a) absent in the negative control (water) which was sampled alongside each experiment; (b) present/identifiable in at least three of the six biological replicates; and (c) the spectrum was matched with confidence to compounds from the NIST17 library. Peaks were then annotated and automatically detected, identified, and integrated using LabSolutions GC‐MS Software (Shimadzu Corporation 1999–2006). The identity of compounds was determined by comparing spectra and retention times between different runs and integration was adjusted manually where necessary. Compound names should be regarded as indicative as these were not verified using authentic standards (see Appendix S2 for spectral data match). Compound levels were analyzed semi‐quantitatively in equivalence to the internal standard (standardized peak area, SPA). Initially, a dilution series was prepared from one replicate per sample to assess the nature of compounds collected from the headspace, their probability of identification, peak separation and to approximate linearity and limit of quantification. An appropriate dilution (strawberry 1:8, plum 1:8, apple 1:16) was determined for each fruit type based on this initial dilution series yielding reasonable linearity (*R* ^2^ > 0.9) for the majority of compounds. Where correlation of the SPA to sample dilution was not possible, volatiles were recorded for presence/absence only.

### 2.5 Statistical analyses of volatile profiles

Chemical diversity was calculated following Simpson's diversity index for proportional data (Hill, 1973) and analyzed using nonparametric tests (Kruskal–Wallis followed by Mann–Whitney U for post hoc analysis, (α = 0.05)). Differences between fruit types, yeast genotypes, and fly attraction were analyzed using a subset of volatiles based on their presence in inoculated fruit of at least two different fruit types. The effects of fruit type and yeast genotype on the chemical composition of the ferments were tested with PERMANOVA as implemented in the R package *vegan* (Anderson, 2001), using Jaccard distances and 10,000 permutations. Constrained correspondence analysis (CCA) was used for data visualization using the full factorial model of *fruit type *× *yeast genotype*. The contribution of each chemical was independently investigated using a full factorial ANOVA design of *fruit type *× *yeast genotype*, including an adjustment for multiple tests (Benjamini and Hochberg method; (Benjamini & Hochberg, 1995). A random forest analysis (Breiman, 2001) was performed using the R package *randomForest* (Liaw, 2002) to identify which compounds correlated with fly attraction and generated bootstrapped regression trees based on the explanatory variables (chemical concentrations) and estimates how important each variable is in explaining the response (AI, fly attraction, treated as a continuous variable). Correlation tests between each individual chemical and the attraction indices of both *D. simulans* and *D. melanogaster* were performed using Pearson's product moment correlation coefficient, and the P‐values were adjusted for multiple tests (Benjamini & Hochberg, 1995). Chemical compounds that were identified as correlating with fly attraction were partitioned using conditional inference tree analysis as implemented in the R package *party* (Horthorn, Hornik, & Zeileis, 2006). Conditional inference trees create binary partitions in the data based on statistically significant differences, minimizing bias and over‐fitting.

## 3 RESULTS

### 3.1 Contrasting yeast preferences in *Drosophila* lines suggest that *D. melanogaster* is likely attracted to a broad range of *Saccharomycetaceae* whereas *D. simulans* response appears species specific

**Figure 2:**
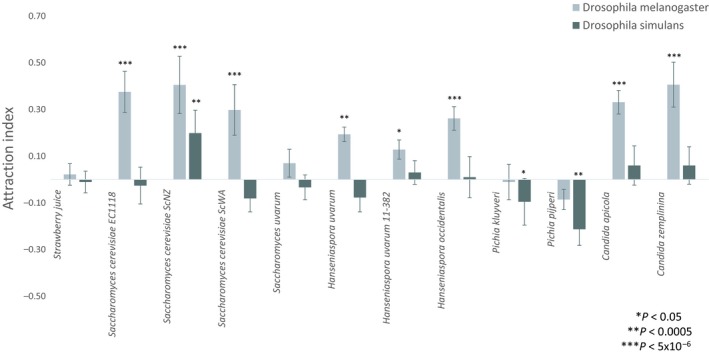
*Drosophila melanogaster* and *Drosophila simulans* attraction to a range of *Saccharomycetaceae* yeasts grown in strawberry juice. Error bars represent the standard error of the mean attraction index and significance in binomial distribution of choice tests is indicated by asterisks (*N* = 6, α = 0.05)

To test whether there are specific yeast preferences between genotypes of *Drosophila* subgroup *“melanogaster”* fly lines, we measured the AI of eleven fruit or insect derived *Saccharomycetaceae* yeast isolates (Table SMM in Appendix S1) grown in strawberry juice using binary choice tests. The *D. melanogaster* line was significantly attracted to eight of the eleven yeasts isolates (Figure 2) spanning *Hanseniaspora uvarum, H. occidentalis*, *Candida zemplininia, C. apicola,* and *S. cerevisiae* (*p* < 0.02, Figure 2). There was no choice preference for *S. uvarum* (*p* = 0.09) or the *Pichia* isolates (*p* > 0.4), and none of the yeast isolates were repulsive to *D. melanogaster*. Using the same yeast ferments, the *D. simulans* line was significantly attracted to just one *S. cerevisiae* strain (ScNZ, *p* = 2x10^‐4^): the same strain previously reported as attractive to this same *D. simulans* line when grown in grape juice (Buser et al., 2014; Günther et al., 2015). However, in contrast to *D. melanogaster*, *D. simulans* was indifferent to most other yeasts, including other *S. cerevisiae* strains, and repulsed by the *Pichia kluyverii* (*p* = 0.02) and *P. pijperi* (*p* = 1 × 10^−4^) isolates (Figure 2). Overall, only one yeast strain was consistently significantly attractive to both fly species (ScNZ), and thus these results support that yeast preference differs, at least between the *Drosophila* genotypes derived from closely related species tested here.

### 3.2 Yeast attraction was stable across fruit types for *D. melanogaster* but *D. simulans* showed context‐dependent behavior

**Figure 3:**
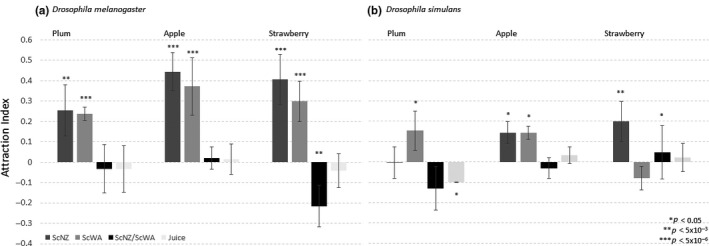
Attractiveness of *Saccharomyces cerevisiae* strains ScNZ and ScWA to *Drosophila melanogaster* (a) and *Drosophila simulans* (b) when inoculated in sterile fruit juice and compared against juice of the same fruit type as control. Binary competition experiments (ScNZ/ScWA) test ferments of both yeasts against each other, where a positive Attraction index (AI) indicates preference for ScNZ and a negative AI preference for ScWA. Error bars represent the standard error of the mean AI and significance in binomial distribution of choice tests is indicated by asterisks (*N* = 6, α = 0.05)

The attraction of both *Drosophila* lines to two *S. cerevisiae* isolates (ScNZ and ScWA (Günther et al., 2015, Buser et al., 2014)) when grown in strawberry, plum, and apple juice showed that this *D. melanogaster* genotype was significantly attracted to both yeast isolates in all fruits (*p* < 7.2 × 10^−5^; Figure 3a) compared to sterile juice. In contrast, whether yeasts were attractive to the *D. simulans* genotype was contingent on the fruit context (Figure 3b). For example, *D. simulans* was attracted to ScNZ in strawberry juice (*p* = 2 × 10^−4^), but not in plum (*p* = 0.5). This same line has previously been shown to be repulsed by ScWA in grape (Günther et al., 2015), and here ScWA had a negative AI in strawberry (AI: −0.08, *p* = 0.12), but was significantly attractive in plum (*p* = 0.002) and apple (*p* = 0.01). Thus, yeast preference of this *D. simulans* genotype was heavily dependent on the fruit environment. Competition (head‐to‐head) comparisons between both yeasts in the various fruits confirmed that *D. melanogaster* was equally attracted to both isolates across fruits except strawberry where ScWA was preferred over ScNZ (*p* = 1.2 × 10^−5^, Figure 3a). Overall, these data support a lack of fruit effect for *D. melanogaster* attraction to yeasts and suggest that any resulting interactions are likely broad and stable. For the *D. simulans* isolate, however, these data indicate that the fruit context significantly affects whether a particular yeast isolate is attractive and therefore any resulting yeast–fly interactions are contingent on both fruit and yeast type, and thus are not generally yeast‐type specific.

### 3.3 *Drosophila* Yeast preferences evolve over ecological time frame

**Figure 4:**
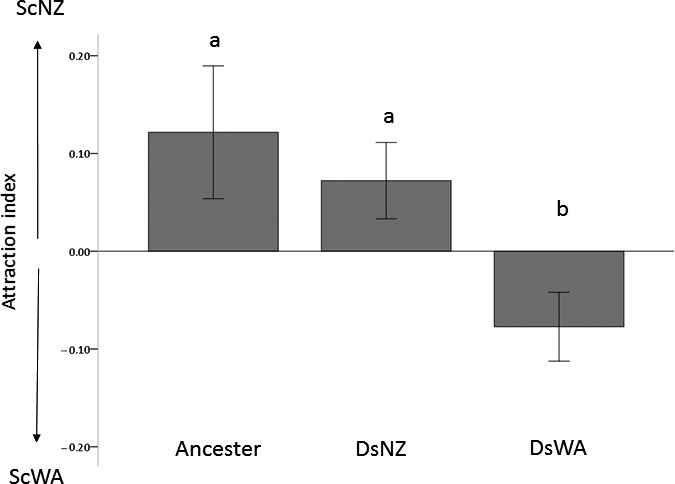
Change in *Drosophila simulans* preference to *Saccharomycetaceae cerevisiae* isolates ScNZ and ScWA in strawberry juice over ten fly generations. Error bars represent the standard error of the mean attraction index (AI, *N* = 6) for ancestral *Drosophila simulans* and lines selected for attraction to ScNZ (DsNZ) or ScWA (DsWA), respectively. Positive AI indicate preference for ScNZ and negative AI attraction to ScWA and treatments not connected by the same letter are significantly different (ANOVA, Tukey HSD test at α = 0.05)

Whether *Drosophila* attraction to particular yeasts is conserved or malleable was examined using experimental evolution of isogenic *D. simulans* over a six‐month period with initially attractive (ScNZ, AI: 0.2; *p* = 0.0002) and less‐preferred yeast (ScWA, AI: −0.05, *p* = 0.01 in head‐to‐head competition with ScNZ, Figure 3b). There was no change in fly preference in the control lines compared to the ancestor (*p* = 0.55, Table SA2 in Appendix S1), showing the experimental system the flies were subjected to had no effect on their yeast preference. However, there were significant differences in fly preferences between the ancestor and lines subject to selection (ANOVA, *F*‐ratio = 5.3, *p* = 0.009) as shown in Figure 4. Tukey HSD post hoc analysis (α = 0.05) show lines selected for attraction to the originally preferential yeast strain (ScNZ) retained this attraction (*p* = 0.87, Table SA3 in Appendix S1). However, fly lines selected for preference to the originally less preferred yeast (ScWA) evolved to become significantly attracted to ScWA (*p* = 0.02, Figure 3, Table SA3 in Appendix S1). Thus, *D. simulans* preference significantly changed over a relatively short timescale to become attracted to the originally less‐preferable yeast strain, with the trade‐off of decreased preference to the originally attractive yeast.

### 3.4 A universal chemical signal driving yeast attraction across *Drosophila* genotypes is unlikely

Thus far, the data show that yeast–fly interactions are not conserved between the *Drosophila* lines tested here and may change between fruit contexts and over time. However, *Drosophila* is able to recognize some component/s of YVOCs and this induces a behavioral response. We tested whether there was a general chemical signal mediating *Drosophila* attraction. Between 41 and 60 volatile compounds were tentatively identified in sterile plum, apple and strawberry juice using GC‐MS, with only 17 compounds common to all fruits. Thus, the majority of volatiles were fruit‐type specific. Fermentation significantly reduced the number of volatiles from an average of 53 ± 6 to 42 ± 4 (*p* = 0.02) but increased profile similarity across different fruits from 13.5% to 68%. The volatile composition between ferments of different fruits had greater similarity to one another than to the sterile juices of those fruits (strawberry: 5.1%; plum: 12.1%; apple: 10.2%). Consequently, fermentation significantly (*p* < 0.001) affected the volatile profile of each fruit type by reducing chemical diversity compared to juice (SC1, Figure SC1 in Appendix S1) and increasing the abundance of single compounds. While it is well known that yeast metabolism alters the composition and concentrations of fruit volatiles (Cordente et al., 2012), we show that the yeast manipulated chemical signatures of ferments from different fruits tend to converge.

The *D. melanogaster* laboratory strain was highly attracted to *S. cerevisiae* ferments regardless of yeast isolate or fruit type, and thus one might conclude that compared to sterile juice, YVOCs unique or predominant in these ferments comprise a common signal and drive attraction. Forty‐two compounds were common in these fruit types and respective ferments and were grouped into those that were as follows: (a) exclusive to juice; (b) exclusive to ferments; and (c) present in both but significantly greater in ferments (Table 1). We found that seven volatiles were indicative of sterile juice. These compounds do not correlate with and thus unlikely represent drivers of fly attraction. Twenty‐six compounds were defined as YVOCs as these were either exclusive to or consistently and significantly (*p* < 0.03) increased in ferments across all samples (Table 1, Appendix S2). Of these, 2‐methylbutanol, 3‐methylbutanol, and 3‐methylbutyl acetate showed a strong interaction between fruit and yeast type (ANOVA with FDR, Table SD1 in Appendix S1) indicating that different yeast isolates are metabolizing fruit precursors in different ways. Of those compounds that did not show a significant interaction, unknown volatile 1 and 2, linalool and β‐damascenone were differentiated between fruit types, but not yeast types, and 1‐heptanol was differentiated between yeast but not fruit types (Table SC1 in Appendix S1).

**Table 1:** Volatiles common to sterile strawberry (S), apple (A) and plum (P) juice and to juice inoculated with *Saccharomyces cerevisiae* strains ScNZ and ScWA, respectively

S	A	P	Detected in fermented juice only	ScNZ	ScWA	Increased in fermented juice
✓_ScWA_	✓_ScWA_	✓_ScWA_	3‐hydroxybutanone (Acetoin)°	↑28x**	↑39x**	1‐propanol
✓	✓	✓	2,4,5‐trimethyl−1,3‐dioxolane A°	↑6x**	↑7x**	2‐pentanone (Methylpropyl ketone)
✓_ScWA_	✓_ScWA_	✓_ScWA_	2,4,5‐trimethyl−1,3‐dioxolane B°	↑65x**	↑170x**	Ethyl propanoate
✓	✓	✓	2‐methylethyl propanoate (Isopropyl propanoate)	↑11000x**	↑8000x**	3‐methyl butanol (Isoamyl alcohol)
✓	✓	✓_ScNZ_	3‐methyl−1‐pentanol	↑18x**	↑25x**	2‐methyl butanol (active Amyl alcohol)
✓	✓	✓	Unknown 2	↑3x*	↑5x**	Ethyl butanoate
✓	✓	✓	Unknown 3	↑2x**	↑3x**	1‐hexanol
✓	✓	✓	methyl 2‐hydroxy−4‐methyl pentanoate	↑29x**	↑25x**	3‐methylbutyl acetate (Isoamylacetate)
✓	✓	✓	2‐methylthiolan−3‐one (Blackberry Thiophenone)	↑2x**	ns	Ethyl acetate
✓	✓	✓	1‐heptanol	↑56x*	ns	2‐methyl−1‐propanol (Isobutanol)
✓	✓	✓	2‐phenylethanol (Benzeneethanol)	ns	↑72x**	Ethyl hexanoate (Ethyl caproate)
✓	✓	✓	ethyl octanoate (Ethyl caprylate)	ns	↑460x*	Octanoic acid (Caprylic acid)
✓	✓	✓	2‐phenethyl acetate	ns	↑10x**	Nonanoic acid (Pelargonic acid)

ScNZ	ScWA	Reduced in fermented juice	ScNZ	ScWA	Unchanged
↓13x**	↓7x**	1‐butanol	ns	ns	Unknown 1
↓11x*	↓21x*	**A:** 2‐methylbutyl acetate (amyl acetate)	ns	ns	2‐methylpropyl acetate (Isobutyl acetate)
↓48x**	↓27x**	1‐hexanal	ns	ns	3‐hexenol
↓11x*	↓8x*	**P**: 1‐(2,6,6‐trimethyl−1,3‐cyclohexadien−1‐yl)−2‐ buten−1‐one (β‐damascenone)	ns	ns	Hexyl acetate
			ns	ns	3,7‐Dimethyl−1,6‐octadien−3‐ol (Linalool)
			ns	ns	2,3‐butanedione (Diacetyl)

Notes

Grouping indicates volatiles that were significantly increased and decreased (*p < 0.05, ***p < 0.005; multivariate analysis of variance, Tukey HSD corrected) in juice compared to ferments. ° indicates that compound levels were outside the quantification limit and only considered for qualitative analysis.

**Figure 5:**
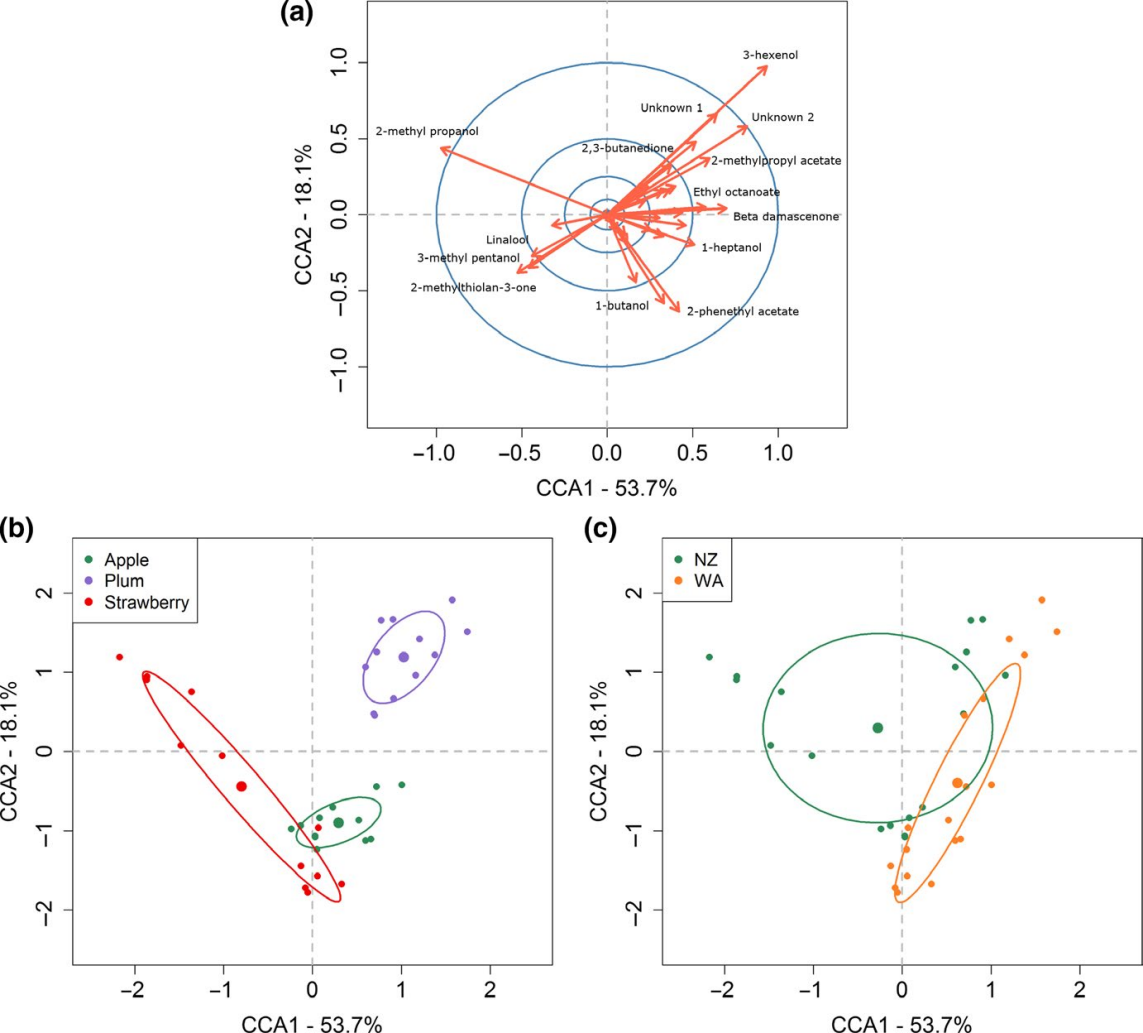
Constrained Correspondence Analysis visualization of fruit ferments using the chemicals listed in Table 1. (a) The direction and magnitude of all loading vectors, with labels for chemicals that report a magnitude larger than 0.5. The blue circles represent the position of 0.1, 0.25, 0.5 and 1 for reference. (b) Sample points colored by fruit type with 50% ellipses. (c) Samples point colored by yeast genotype with 50% ellipses

A clear separation between fruit type (Figure 5b) and yeast genotype (Figure 5c) was visualized using CCA, and three compounds (3‐hexenol, 2‐methyl propanol, and unknown 2) had loadings (Eigenvectors * √Eigenvalues) with the highest magnitude in the analysis (Figure 5a), thus appearing to drive variation between samples.

An alternative approach to analyze the multivariate chemical profiles uses PermANOVA and this confirmed that volatile profiles were affected by both yeast (*R* ^2^ = 9%, *p* = 1 x 10^−4^) and fruit type (*R* ^2^ = 19%, *p* = 1 x 10^−4^, Table SE2 in Appendix S1). However, the interaction between fruit and yeast type explained 27% (*p* = 1 x 10^−4^) of the total variation indicating, in line with the preceding approach, that YVOCs are the likely product of a complex, metabolic interaction between different fruits and yeasts.

The correlation between volatile profiles and fly attraction was further analyzed by integrating the AI's (Table SE1 in Appendix S1) with the volatile data using a random forest analysis. Analyses of both *D. simulans* and *D. melanogaster* returned best models where the predicted AI of each fly was negatively correlated with the observed AI, indicating models were not able to identify a significant effect of single compounds on fly attraction. After adjusting the *P*‐values for multiple tests, there were no significant correlations with individual compounds and AI (Table SE1 in Appendix S1). This shows that single compounds are not strongly implicated in fly attraction. However, relying on *p*‐values solely may be misleading particularly when multiple tests are performed (Krzywinski & Altman, 2014). To further interrogate the data, we went on to use conditional inference tree analysis: for *D. simulans*, significant binary splits were made for ethyl acetate, 3‐methyl butanol, and 2‐methyl butanol (Figure SF1 in Appendix S1), implicating these in attraction. Greater ethyl acetate and 2‐methyl butanol concentrations correlate with a greater attraction response (*p* = 0.04 and *p* = 0.01) for *D. simulans* as part of a blend when present above defined thresholds. When inoculated in strawberry juice, the levels of six YVOCs were significantly (Mann–Whitney *U*‐test, *p* < 0.05, Table SC4 in Appendix S1) increased in the preferred yeast ScNZ compared to ScWA ferments and these included ethyl acetate, 3‐methyl butanol, 2‐methyl butanol besides 2‐methyl propanol, hexyl acetate, and 2‐phenyl ethanol. For *D. melanogaster*, a significant binary split could be made for 2‐methylpropyl acetate, a compound that also differs between fruit‐type. Here, attraction decreased with greater 2‐methylpropyl acetate levels suggesting a repulsive effect above thresholds (Figure SF2 in Appendix S1). Given that Conditional Interference trees was the only test that showed any significant result, further experiments would be required to verify these findings. Overall, there is no compelling data to support the hypothesis that specific yeast volatiles might act as general signal driving attraction of *Drosophila*. While the data confirm an array of YVOCs as candidates for attraction of the *D. melanogaster* line, flies from the *D. simulans* population were likely to respond in a context‐specific and threshold‐dependent way.

## 4 DISCUSSION

If selection has been and is operating on chemically mediated interactions between *Drosophila* and *Saccharomycetaceae* yeasts, then one predicts specific yeast genotypes to be consistently attractive (or repulsive). The data categorically reject this general hypothesis, with only one (ScNZ) of eleven yeast isolates demonstrating consistency in attractiveness to both *Drosophila* tested. Instead the data show *Drosophila*‐genotype‐specific profiles where *D. simulans* was indifferent to, and *D. melanogaster* was attracted to, the majority of yeast isolates (Figure 2). This in turn suggests that either specific yeast preferences evolved de novo for each of these isolates, or any intrinsic response inherited from the common *D. simulans*/*melanogaster* ancestor to have differentially evolved.

Spatial or temporal resource partitioning is a commonly described mechanism promoting coexistence of species using the same nutritional resource (Chesson, 2000). For example, the Drosophilids *Zaprionus indianus* and *D. simulans* coexist in figs by colonizing the fruit at different ripening stages and larval dietary requirements of both species correspond to increasing yeast infestation during fruit ripening (Matavelli, Carvalho, Martins, & Mirth, 2015). This raises the question whether differentiation in chemosensory preference might promote the coexistence of closely related species. *D. simulans* and *D. melanogaster* are evolutionary siblings that split about 2.5 million years ago (Clark et al., 2007), form hybrids and live in sympatry. Possible mechanisms regulating their coexistence were mainly explained by adaptation strategies leading to seasonal variation in life history traits and differences in their ecophysiology (Capy & Gibert, 2004; David et al., 2004). Some studies also report increased ethanol and concomitant acetic acid sensitivity in *D. simulans* (Chakir, Peridy, Capy, Pla, & David, 1993). However, the degree to which differential preferences for microbial volatiles may have impacted resource selection and thus niche differentiation between the two species has to our knowledge not been previously investigated, and these data suggest this possibility. The caveat to this conclusion is that single lines of flies were used—it would be valuable to understand the variance in attraction among different fly genotypes in *D. simulans*/*melanogaster* generally.

Following the logic introduced above, if selection has been and is operating on YVOC‐mediated specific *Drosophila* attraction then maintenance of any associated traits would be expected regardless of environmental factors like the fruit context. The data support this hypothesis for the *D. melanogaster* line where both *S. cerevisiae* isolates were significantly attractive in all three fruits (Figure 3a) but not for *D. simulans*’ yeast preference which was contingent on the fruit host. This shows the fruit context modulates yeast attraction in one fly line only, and that response again differs between *Drosophila* genotypes which provides further support that resulting yeast–*Drosophila* interactions may evolve readily.

We tested whether this trait was movable or stable directly, using experimental evolution with *D. simulans* and show that fly preference significantly changed over ten generations to become attracted to an originally less‐preferable yeast (Figure 4). The generation time of *Drosophila* in nature is not clear, but the ten generations/six months in controlled conditions covered in this experiment likely represent at least a summer season in the wild. Thus, the attraction of the *D. simulans* isolate to specific yeasts appears to be plastic and capable of fluctuating over short ecologically representative time periods, even within members from an isogenic female population. Although the nature of this heritable change is unknown, these data reject the hypothesis that at least *D. simulans'* yeast preference is conserved. It suggests that selection may operate and readily move this trait, possibly by affecting sensitivity to particular YVOCs which could be genetic, epigenetic, or due to shifts in commensal microbiota. This is a third line of evidence providing support that yeast–*Drosophila* interactions are potentially capable of rapidly evolving.

That yeast fermentation changes volatile profiles of fruits is well known. However, it is surprising to find such a high degree of volatile homogeneity across ferments from vastly different horticultural crops, indicating a universal chemical signal for the presence of yeasts. The majority of these YVOCs were tentatively classified as esters (in particular ethyl and acetate esters), fusel‐alcohols, and other intermediates of the Ehrlich‐pathway (Günther & Goddard, 2019; Hazelwood et al., 2008). In particular, C_2_/C_3_ substituted or branched‐chained volatiles likely resulting from amino acid assimilation were abundant. *Drosophila* odor receptors are known to respond to these metabolites, in particular 3‐hydroxy‐2‐butanone, 2‐phenyl ethanol, 2‐phenethyl acetate, 3‐methyl butanol, and 3‐methylbutyl acetate were previously suggested to act as key compounds for chemical attraction (Arguello, Sellanes, Lou, & Raguso, 2013; Becher et al., 2012; Günther et al., 2015; Stökl et al., 2010), and it is likely these volatiles are involved in driving attraction in *D. melanogaster*. However, *D. simulans’* attraction was sensitive to the fruit, threshold and context dependent. It appears that flies might respond to structurally related compounds rather than to individual ones but what defines these mechanisms or drives them apart between species is yet to be elucidated.

Overall, this suite of experiments strongly suggests that chemically mediated interactions differ at least between individual lines of *D. melanogaster*/*D. simulans* and *Saccharomycetaceae* yeasts. Further, the strength and nature of the *D. simulans*–yeast attraction may change over relatively short ecological timescales. At the least, these data strongly suggest that the evolution of chemosensory perception differs between two *Drosophila* genotypes in the *melanogaster* subgroup, and thus supports a conclusion that there is no ancient or “fixed” aspect to *Drosophila* (subgroup *melanogaster)*–yeast interaction that selection has maintained. The data do support the conclusion that the association between some *Drosophila* lines (in this case *D. melanogaster)* and *Saccharomycetaceae* yeasts is relatively stable. However, the chemical mechanisms of this interaction remain elusive. Overall, there is no strong evidence to support the idea that there are bespoke interactions with specific yeast species or strains for either of these organisms, rather the data support the idea that *Drosophila* are generally adapted to sense and locate fruits infested by a range of fungal microbes, as would be expected in nature. While particular experiments with interspecies interactions may appear to conform to mutualisms, it is perhaps necessary to understand the variation of these traits among populations and species to fully substantiate any claims as to whether natural selection has produced adaptations for mutualistic interactions.

We postulate these interactions may be due to two possible reasons. Firstly, they may be due to exaptation (selection operated on yeast volatile production for other reasons), and that flies have evolved to sense YVOC's to prey on yeasts, in which case specific yeast transfer between fruits by *Drosophila* and other insects is nothing greater than an fortuitous event, and not one driven by natural selection (Günther & Goddard, 2019). Alternatively, the ecological reality is that microbes exist as communities (a mix of different individuals and species) in/on fruit (Taylor et al., 2014), and the concept of a single strain of any microbe metabolizing and emitting volatiles in isolation seems highly unlikely. It is possible that selection may instead operate on a higher‐level “community bouquet.” Here, selection operates on fly attraction to YVOCs produced by more general yeast (and possibly bacterial) infected fruits, and that this microbial community is then generally dispersed to other fruits. This hypothesis predicts that mixes of yeast strains and species will be more attractive than individual isolates, and that selection will have operated to assemble a core set of species that comprise a fruit microbiome.

## CONFLICT OF INTERESTS

The authors declare no competing financial or non‐financial interests.

## AUTHORS’ CONTRIBUTIONS

CSG and MRG conceived and designed the experiments; CSG and RJ performed the experiments; CSG, SJK, RJ, and MG analyzed the data; CSG, SJK, and MRG wrote the manuscript.

## Data Availability

The datasets generated and/or analyzed during the current study are publicly available through the University of Lincoln Data repository (http://eprints.lincoln.ac.uk/36052/).
