# Supplementary material for: Are Drosophila preferences for yeasts stable or contextual?
Source: Ecol Evol. 2019 Jun 30;9(14):8075–86. doi: 10.1002/ece3.5366 (PMC6662392; doi:10.1002/ece3.5366)
Supplement: Supplementary file 1 [file ECE3-9-8075-s001.pdf]

*Supplemental material: "***Are** ***Drosophila*****-preferences for yeasts stable or contextual?***"*

by Catrin S. Günther, Sarah J. Knight, Rory Jones and Matthew R. Goddard

Table SMM: Origin of *Saccharomycetaceae* isolates.

Name	Country of origin	Source	Reference
Saccharomyces cerevisiae 'Lalvin' EC-1118	France	Commercial wine yeast	Lallemand inc. Lallemandbrewing.com
Saccharomyces cerevisiae Fly_KR78.3	New Zealand	*Drosophila simulans*	Buser et al 2014. Ecology Letters 17: 157-64
Saccharomyces cerevisiae DBVP6044	West Africa	Bili Wine	Liti et al. 2009. Nature 458: 337-41
Saccharomyces uvarum SBJ1d	New Zealand	Sauvignon Blanc ferment	Goddard culture collection
Hanseniaspora uvarum 11-382	USA	*Drosophila suzukii*	Phaff Yeast culture collection, UC-Davis
Hanseniaspora uvarum HB-62	New Zealand	Chardonnay fruit	Gayevskiy et al. 2012. ISME Journal 6:1281-90
Hanseniaspora occidentalis WI-82	New Zealand	Syrah fruit	
Pichia kluyverii JT3.71	New Zealand	Chardonnay juice	Anfang et al. 2009. Australian Journal of Grape and Wine Research 15:1-8
Pichia pijperi	New Zealand	Pinot noir ferment	Goddard culture collection
Candida apicola X120705S2.1	New Zealand	Beehive	Anfang et al. 2009. Australian Journal of Grape and Wine Research 15:1-8
Candida zemplininia FA2.12	New Zealand	Chardonnay ferment	

Supplemental A

Data were analysed using IBM SPSS Statistics Version 21

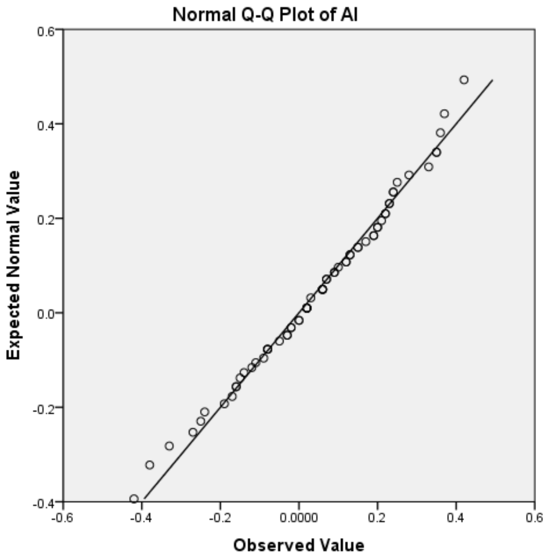

Figure SA1: Q-Q plots indicating roughly normal distribution of combined attraction Indices (AI, N=66) testing *Drosophila simulans* (ancestral line/F0, 3 independent F11 populations) preference for *Saccharomyces cerevisiae*_ ScNZ against *S. cerevisiae* ScWA in two-way choice tests.

TableSA1: Levene's Test of Equality of Error Variances. Tests the null hypothesis of homogeneity of variances in Attraction indices (AI) as the dependent variable across *Drosophila simulans* populations.

F	df1	df2	Sig.
.494	3	62	.687

TableSA2: Paired Samples Test. Testing the null hypothesis that there is no difference in *Drosophila simulans* attraction (AI as dependent variable) for *Saccharomyces cerevisae* ScNZ between the ancestral line (DsF0) and evolved (DsNZF11, DsWAF11) or control/ unevolved offspring population (DsCF11).

		Mean	Std. Deviation	Std. Error Mean	95% Confidence Interval of the Difference		t	df	Sig. (2-tailed)
					Lower	Upper			
Pair 1	DsF0 - DsCF11	.05500	.30480	.08799	-.13866	.24866	.625	11	.545
Pair 2	DsF0 - DsNZF11	.07417	.27550	.07953	-.10088	.24921	.933	11	.371
Pair 3	DsF0 - DsWAF11	.22833	.30522	.08811	.03440	.42226	2.591	11	.025

TableSA3: Tukey HSD corrected Multiple Comparisons Testing the null hypothesis that there is no difference in *Drosophila simulans* attraction (AI as dependent variable) for *Saccharomyces cerevisae*_ScNZ between each fly population. DsF0: ancestral line; DsNZF11: evolved to select *S. cerevisiae*_ScNZ; DsWAF11: evolved to select *S. cerevisiae*_ScWA; DsCF11 control/ unevolved offspring

Population (I)	(J) Population	Mean Difference (I-J)	Std. Error	Sig.	95% Confidence Interval	
					Lower Bound	Upper Bound
DsF0	DsCF11	.0161	.06528	.995	-.1562	.1884
	DsNZF11	.0494	.06528	.873	-.1229	.2218
	DsWAF11	.1989*	.06528	.017	.0266	.3712
DsF11	DsF0	-.0161	.06528	.995	-.1884	.1562
	DsNZF11	.0333	.05838	.940	-.1208	.1875
	DsWAF11	.1828*	.05838	.014	.0286	.3369
DsNZF11	DsF0	-.0494	.06528	.873	-.2218	.1229
	DsCF11	-.0333	.05838	.940	-.1875	.1208
	DsWAF11	.1494	.05838	.061	-.0047	.3036
DsWAF11	DsF0	-.1989*	.06528	.017	-.3712	-.0266
	DsCF11	-.1828*	.05838	.014	-.3369	-.0286
	DsNZF11	-.1494	.05838	.061	-.3036	.0047

Based on observed means.
The error term is Mean Square(Error) = .031.

*. The mean difference is significant at the .05 level.

Supplemental C

Table SC1: Number of different peaks, detected in the headspace of sterile and yeast- inoculated juice

Fruit type	sample	N volatiles
Strawberry	Yeast ScNZ	46
	Yeast ScWA	44
	juice	60
Plum	Yeast ScNZ	41
	Yeast ScWA	44
	juice	49
Apple	Yeast ScNZ	35
	Yeast ScWA	41
	juice	51

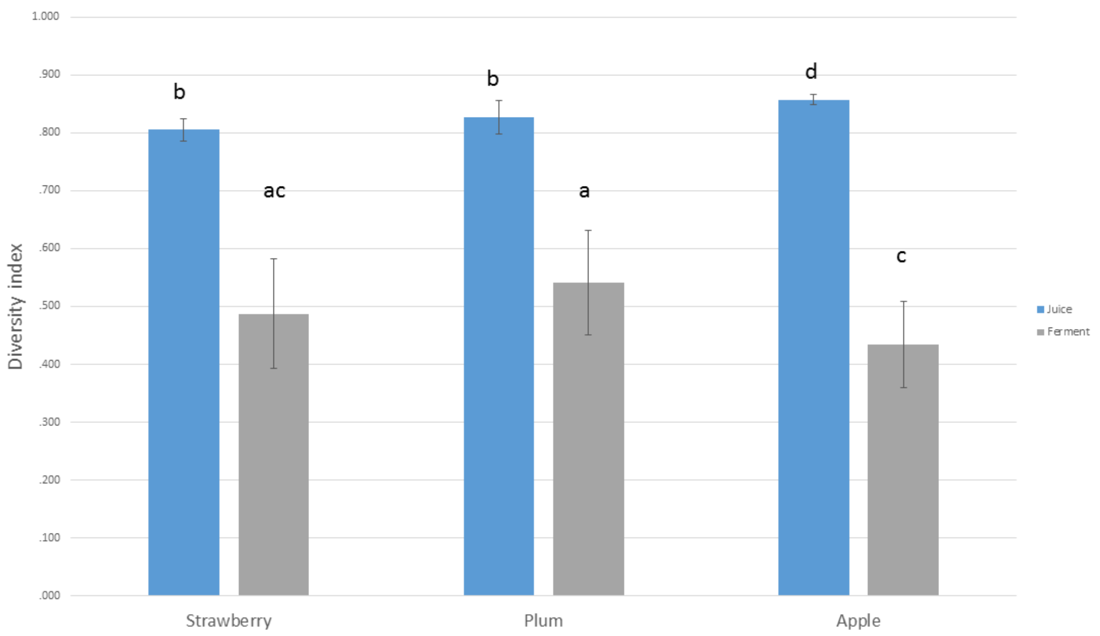

FigSC1: Chemical diversity of headspace volatiles from inoculated (blue) and sterile (grey) juice. The error bars represent the standard deviation of the mean diversity index (Simpson's diversity) reflecting counts and concentrations of chemical compounds. Kruskal Wallis-H test (X^2=40.4; p<0.0001) indicates a significantly different diversity distribution between samples. This has been confirmed for pairwise comparison using the Mann-Whitney U test, except for samples highlighted with the same letters which are not significantly different from each other in their median chemical diversity.

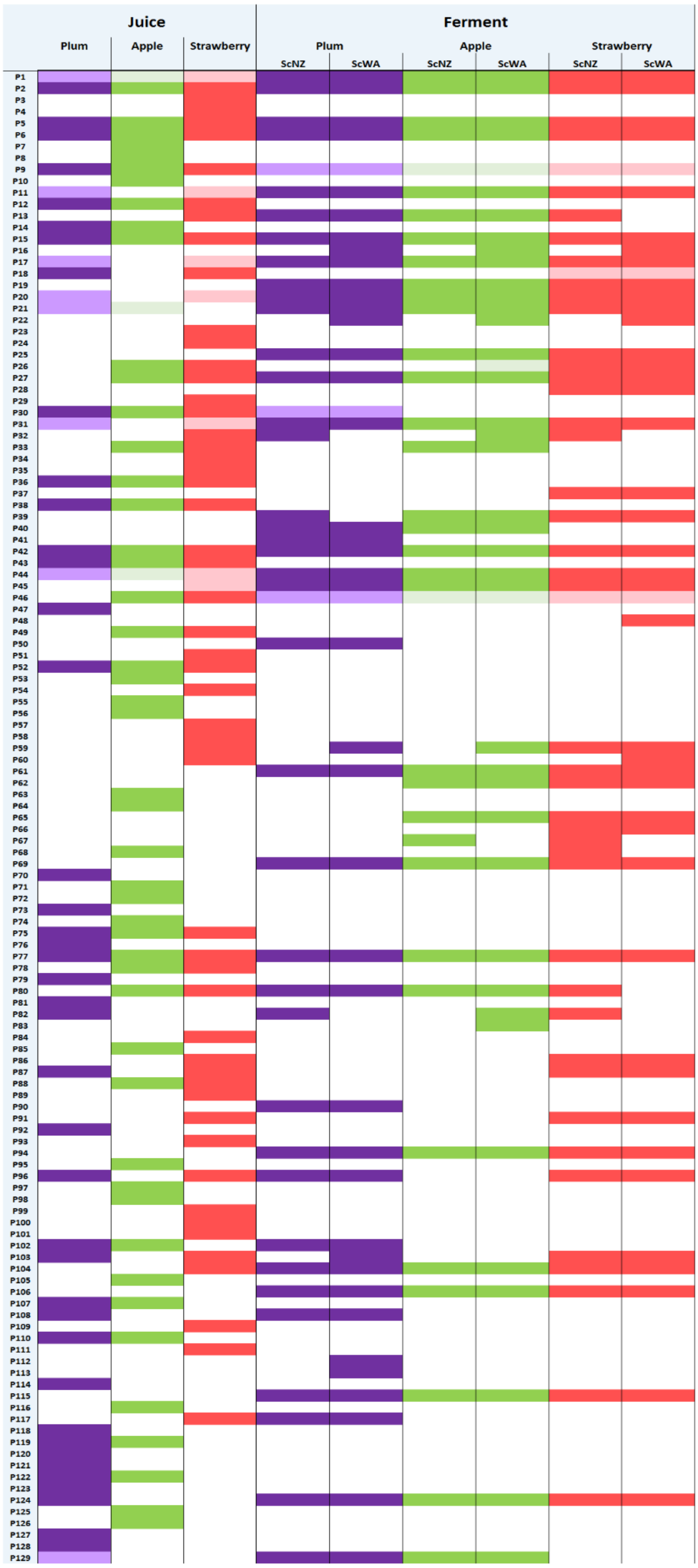

Figure SC2: Volatile profiles of sterile fruit juice and after inoculation with *Saccharomyces cerevisiae* strain ScNZ and ScWA, respectively. Increased colour transparency indicates that compound levels were significantly ($P<0.05$) lower when compared to the sterile or inoculated sample of the corresponding fruit (TukeyHSD-corrected Manova, see TableSD1 and SD2).

TableSC2: Fruit-specific and yeast specific compounds detected in sterile plum (Pl), apple (Ap) and strawberry (St) juice and after inoculation with *Saccharomyces cerevisiae* ScNZ and *S. cerevisiae* ScWA, respectively. ✓ indicates that the compound is present in a sample and x that is was not detected.

		Juice			ScNZ			ScWA		
**Name**	**Peak**	**Pl**	**Ap**	**St**	**Pl**	**Ap**	**St**	**Pl**	**Ap**	**St**
1-penten-3-ol	**P12**	✓	✓	✓	x	x	x	x	x	x
3-hydroxy-2-butanone	**P16**	x	x	x	x	x	x	✓	✓	✓
2,4,5-trimethyl-1,3-dioxolane A	**P19**	x	x	x	✓	✓	✓	✓	✓	✓
2,4,5-trimethyl-1,3-dioxolane B	**P22**	x	x	x	x	x	x	✓	✓	✓
2-methylethyl propanoate	**P25**	x	x	x	✓	✓	✓	✓	✓	✓
hexanal	**P30**	✓	✓	✓	✓	x	x	✓	x	x
2-hexenal A	**P36**	✓	✓	✓	x	x	x	x	x	x
2-hexenal B	**P38**	✓	✓	✓	x	x	x	x	x	x
3-methyl pentanol	**P39**	x	x	x	✓	✓	✓	x	✓	✓
Unknown 2	**P40**	x	x	x	✓	✓	✓	✓	✓	✓
2-hexen-1-ol	**P43**	✓	✓	✓	x	x	x	x	x	x
heptanal	**P52**	✓	✓	✓	x	x	x	x	x	x
NoID	**P61**	x	x	x	✓	✓	✓	✓	✓	✓
methyl 2-hydroxy-4-methylpentanoate	**P62**	x	x	x	✓	✓	✓	✓	✓	✓
2-methylthiolan-3-one	**P65**	x	x	x	✓	✓	✓	✓	✓	✓
1-heptanol	**P69**	x	x	x	✓	✓	✓	✓	✓	✓
butyl butanoate	**P75**	✓	✓	✓	x	x	x	x	x	x
2-phenylethanol	**P94**	x	x	x	✓	✓	✓	✓	✓	✓
Ethyl octanoate	**P106**	x	x	x	✓	✓	✓	✓	✓	✓
2-phenethyl acetate	**P115**	x	x	x	✓	✓	✓	✓	✓	✓

TableSC3 Multivariate analysis of volatiles quantified from sterile and inoculated juice (*Saccharomyces cerevisiae* ScNZ and *S. cerevisiae* ScWA, N=6) in plum, apple, strawberry. MANOVA was employed and corrected for multiple comparisons using Tukey HSD to test for the null-hypothesis that standard-mean levels of volatiles are equal and yeast has no effect on volatile composition. *P*<0.05 rejects this null hypothesis and indicates that compound levels were consistently different. The asterisk highlights volatiles different in sterile compared to inoculated juice across all fruit types.

<table>
<tr><th></th><th></th><th colspan="3">P (Juice-ScNZ)</th><th colspan="3">P(Juice-ScWA)</th><th colspan="3">P(ScNZ-ScWA)</th></tr>
<tr><th>Name</th><th>Peak</th><th>Pl</th><th>Ap</th><th>St</th><th>Pl</th><th>Ap</th><th>St</th><th>Pl</th><th>Ap</th><th>St</th></tr>
<tr><td>1-propanol</td><td>P1</td><td colspan="3">0.001</td><td colspan="3"><0.001</td><td colspan="3">0.114</td></tr>
<tr><td>2,3-butanedione</td><td>P2</td><td colspan="3">0.077</td><td colspan="3">0.362</td><td colspan="3">0.591</td></tr>
<tr><td>ethyl acetate</td><td>P5</td><td colspan="3">0.001</td><td colspan="3">0.250</td><td colspan="3">0.026</td></tr>
<tr><td>2-methyl propanol</td><td>P6</td><td colspan="3">0.008</td><td colspan="3">0.888</td><td colspan="3">0.009</td></tr>
<tr><td>1-butanol*</td><td>P9</td><td colspan="3"><0.001</td><td colspan="3">0.001</td><td colspan="3">0.964</td></tr>
<tr><td>2-pentanone*</td><td>P11</td><td colspan="3"><0.001</td><td colspan="3"><0.001</td><td colspan="3">0.009</td></tr>
<tr><td>Unknown 1</td><td>P13</td><td colspan="3">0.082</td><td colspan="3">0.305</td><td colspan="3">0.697</td></tr>
<tr><td>ethyl propanoate*</td><td>P17</td><td colspan="3">0.024</td><td colspan="3"><0.001</td><td colspan="3"><0.001</td></tr>
<tr><td>3-methyl butanol*</td><td>P20</td><td colspan="3"><0.001</td><td colspan="3"><0.001</td><td colspan="3">0.054</td></tr>
<tr><td>2-methyl butanol*</td><td>P21</td><td colspan="3"><0.001</td><td colspan="3"><0.001</td><td colspan="3">0.998</td></tr>
<tr><td>2-methylpropyl acetate</td><td>P27</td><td colspan="3">0.952</td><td colspan="3">0.596</td><td colspan="3">0.707</td></tr>
<tr><td>hexanal*</td><td>P30</td><td colspan="3"><0.001</td><td colspan="3"><0.001</td><td colspan="3">0.985</td></tr>
<tr><td>ethyl butanoate*</td><td>P31</td><td colspan="3">0.028</td><td colspan="3"><0.001</td><td colspan="3">0.084</td></tr>
<tr><td>3-hexenol</td><td>P42</td><td colspan="3">0.568</td><td colspan="3">0.579</td><td colspan="3">1</td></tr>
<tr><td>1-hexanol*</td><td>P44</td><td colspan="3">0.001</td><td colspan="3"><0.001</td><td colspan="3">0.016</td></tr>
<tr><td>3-methylbutyl acetate*</td><td>P45</td><td colspan="3"><0.001</td><td colspan="3"><0.001</td><td colspan="3">0.853</td></tr>
<tr><td>2-methylbutyl acetate*</td><td>P46</td><td colspan="3">0.013</td><td colspan="3">0.017</td><td colspan="3">0.997</td></tr>
<tr><td>ethyl hexanoate</td><td>P77</td><td colspan="3">0.104</td><td colspan="3"><0.001</td><td colspan="3"><0.001</td></tr>
<tr><td>linalool</td><td>P96</td><td colspan="3">0.414</td><td colspan="3">0.582</td><td colspan="3">0.947</td></tr>
<tr><td>octanoic Acid</td><td>P104</td><td colspan="3">0.069</td><td colspan="3"><0.001</td><td colspan="3"><0.001</td></tr>
<tr><td>nonanoic acid</td><td>P124</td><td colspan="3">0.072</td><td colspan="3">0.044</td><td colspan="3">0.954</td></tr>
<tr><td>beta damascenone*</td><td>P129</td><td colspan="3">0.023</td><td colspan="3">0.039</td><td colspan="3">0.972</td></tr>
</table>

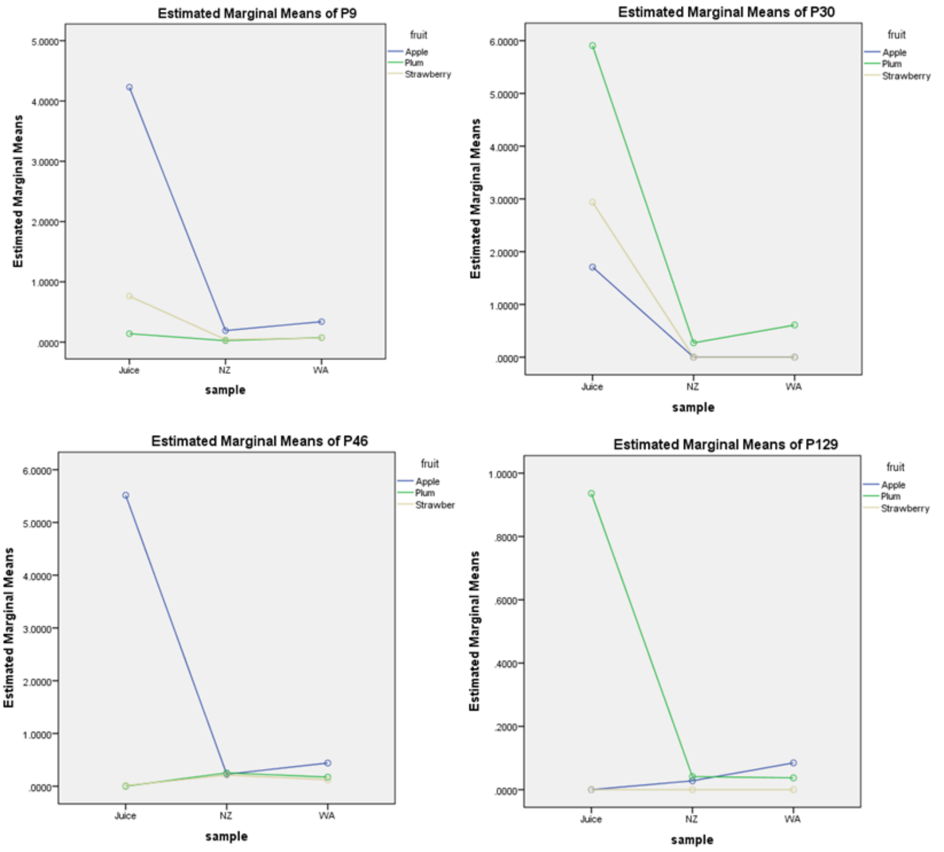

FigureSC3: Volatiles that significantly decreased after inoculation of sterile plum, apple and strawberry juice with *Saccharomyces cerevisiae* Fly_KR78.3 (ScNZ) and *S. cerevisiae* DBVP6044 (ScWA), N=6, TukeyHSD-corrected MANOVA): butanol (P9), hexanal (P30), 2-methylbutyl acetate (P46), β-damascenone (P129).

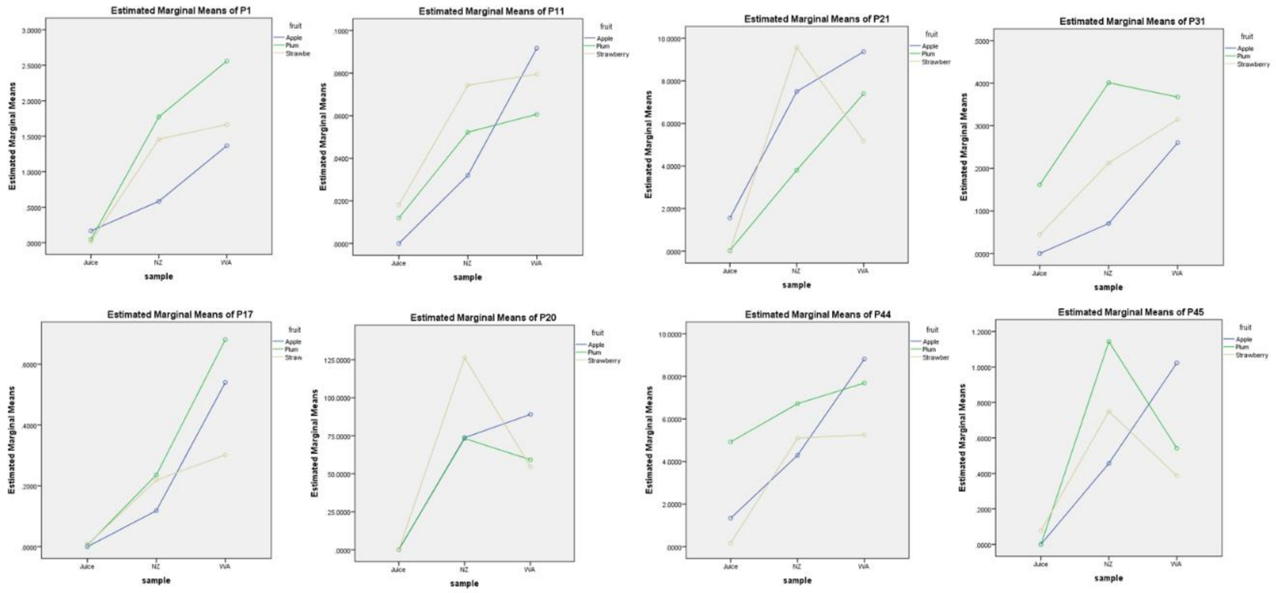

FigureSC4: Volatiles that significantly increased after inoculation of sterile plum, apple and strawberry juice with *Saccharomyces cerevisiae* ScNZ and *S. cerevisiae Sc*WA, N=6, TukeyHSD-corrected MANOVA): Propanol (P1), 2-Pentanone (P11), ethyl propanoate (P17), 3-methylbutanol (P20), 2-methylbutanol (P21), hexanol (P44), 3-methylbutyl acetate (P45).

TableSC4: Non-parametric analysis of yeast volatiles in strawberry ferments using Mann-Whitney U statistics comparing *Saccharomyces cerevisiae* ScNZ and *S. cerevisiae Sc*WA (N=6, α=0.05).

Test Statistics[a]

	P1	P5	P6	P9	P17	P20	P21	P25	P27	P31	P44	P45	P46	P69	P77	P80	P94	P106	P115
Mann-Whitney U	18.000	.000	.000	7.000	9.000	.000	5.000	6.000	7.500	6.000	18.000	7.000	10.000	.000	1.000	.000	.000	4.500	13.000
Wilcoxon W	39.000	21.000	21.000	28.000	30.000	21.000	26.000	27.000	28.500	27.000	39.000	28.000	31.000	21.000	22.000	21.000	21.000	25.500	34.000
Z	.000	-2.882	-2.882	-1.761	-1.441	-2.882	-2.082	-1.922	-1.684	-1.925	.000	-1.761	-1.283	-2.882	-2.722	-3.077	-2.882	-2.166	-.802
Asymp. Sig. (2-tailed)	1.000	.004	.004	.078	.150	.004	.037	.055	.092	.054	1.000	.078	.199	.004	.006	.002	.004	.030	.423
Exact Sig. [2*(1-tailed Sig.)]	1.000[b]	.002[b]	.002[b]	.093[b]	.180[b]	.002[b]	.041[b]	.065[b]	.093[b]	.065[b]	1.000[b]	.093[b]	.240[b]	.002[b]	.004[b]	.002[b]	.002[b]	.026[b]	.485[b]

a. Grouping Variable: group

b. Not corrected for ties.

Supplemental D

TableSD1: Summary of the full factorial ANOVA results for each chemical compound against fruit type and yeast genotype. The P-values have been adjusted for multiple comparisons. F-statistics above 5 and P-values below 0.05 are highlighted in red.

Peak	Compound	Fruit		Yeast		Fruit*Yeast	
		F-stat	*P*-value	F-stat	*P*-value	F-stat	*P*-value
**P1**	1-propanol	11.44	0.001	11.46	0.004	1.67	0.238
**P2**	2,3-butanedione	20.97	0.000	17.22	0.001	18.06	0.000
**P5**	ethyl acetate	11.38	0.001	11.17	0.004	8.59	0.002
**P6**	2-methyl propanol	75.51	0.000	77.98	0.000	83.46	0.000
**P9**	1-butanol	31.32	0.000	8.88	0.010	2.23	0.152
**P11**	2-pentanone	3.42	0.063	13.41	0.002	4.79	0.024

**P13**	Unknown 1	28.81	0.000	1.04	0.345	1.16	0.352
**P17**	ethyl propanoate	4.04	0.041	41.19	0.000	5.53	0.015
**P20**	3-methyl butanol	3.33	0.065	7.55	0.016	12.33	0.000
**P21**	2-methyl butanol	3.94	0.043	0.42	0.537	10.95	0.001
**P25**	2-methylethyl propanoate	2.55	0.119	0.36	0.555	3.06	0.080
**P27**	2-methylpropyl acetate	38.47	0.000	4.50	0.060	10.07	0.001
**P31**	ethyl butanoate	11.58	0.001	6.80	0.022	0.65	0.540
**P39**	3-methyl pentanol	29.22	0.000	0.39	0.540	5.81	0.012
**P40**	Unknown 2	38.53	0.000	1.22	0.315	1.22	0.342
**P42**	3-hexenol	2065.74	0.000	22.78	0.000	6.77	0.007
**P44**	1-hexanol	9.01	0.002	26.33	0.000	10.64	0.001
**P45**	3-methylbutyl acetate	3.37	0.064	1.70	0.237	15.95	0.000
**P46**	2-methylbutyl acetate	7.63	0.004	1.47	0.268	6.55	0.008
**P61**	Unknown 3	4.62	0.027	4.27	0.064	18.41	0.000
**P62**	methyl 2-hydroxy-4-methyl pentanoate	1.29	0.323	10.01	0.006	23.41	0.000
**P65**	2-methylthiolan-3-one	28.93	0.000	3.39	0.097	11.31	0.001
**P69**	1-heptanol	0.92	0.430	117.82	0.000	2.80	0.097
**P77**	ethyl hexanoate	12.51	0.000	51.75	0.000	3.97	0.043
**P80**	hexyl acetate	14.06	0.000	8.33	0.012	26.56	0.000
**P94**	2-phenylethanol	36.17	0.000	10.89	0.005	22.79	0.000
**P96**	linalool	129.37	0.000	0.49	0.510	1.03	0.395
**P104**	octanoic acid	2.20	0.154	47.90	0.000	4.73	0.025
**P106**	ethyl octanoate	16.81	0.000	95.59	0.000	11.42	0.001
**P115**	2-phenethyl acetate	26.98	0.000	20.36	0.000	18.78	0.000
**P124**	nonanoic acid	10.85	0.001	2.33	0.163	12.20	0.000
**P129**	beta-damascenone	10.58	0.001	4.38	0.063	2.50	0.122

TableSD2: Summary of the PERMANOVA results testing the effects of fruit type and yeast strain on the chemical composition of ferments

Factor	Degrees of Freedom	F-statistic	R^2 value	*P*-value
Fruit	2	6.29	0.189	1×10^{-4}
Yeast	1	6.09	0.091	9×10^{-4}
Fruit*Yeast	2	9.02	0.270	1×10^{-4}
Residuals	30		0.450	
Total	35		1	

Supplemental E

TableSE1 Summary of test statistics and P-values of correlation tests between each chemical compound and the attraction index for *Drosophila simulans* and *D. melanogaster*. Marginally non-significant correlations are highlighted in red.

		D. simulans				*D. melanogaster*			
**Peak**	**Compound**	**t-stats**	**r-value**	**p-value**	**adjusted p-value**	**t-stats**	**r-value**	**p-value**	**adjusted p-value**
**P1**	1-propanol	-0.329	-0.056	0.744	0.929	-1.754	-0.288	0.088	0.856
**P2**	2,3-butanedione	1.027	0.174	0.311	0.906	-0.911	-0.154	0.369	0.978
**P5**	ethyl acetate	2.129	0.343	0.041	0.433	1.267	0.212	0.214	0.856
**P6**	2-methyl propanol	1.833	0.300	0.076	0.522	1.012	0.171	0.319	0.978
**P9**	1-butanol	0.536	0.092	0.595	0.929	0.160	0.028	0.873	0.978
**P11**	2-pentanone	0.210	0.036	0.835	0.93	0.608	0.104	0.547	0.978
**P13**	Unknown 1	0.629	0.107	0.534	0.929	-0.098	-0.017	0.922	0.978
**P17**	ethyl propanoate	0.437	0.075	0.665	0.929	-0.553	-0.094	0.584	0.978
**P20**	3-methyl butanol	2.601	0.407	0.014	0.218	0.417	0.071	0.679	0.978
**P21**	2-methyl butanol	2.807	0.434	0.008	0.218	0.185	0.032	0.854	0.978
**P25**	2-methylethyl propanoate	1.517	0.252	0.138	0.554	0.191	0.033	0.850	0.978
**P27**	2-methylpropyl acetate	-0.331	-0.057	0.742	0.929	-2.192	-0.352	0.035	0.856
**P31**	ethyl butanoate	-0.310	-0.053	0.758	0.929	-1.373	-0.229	0.179	0.856
**P39**	3-methyl pentanol	0.765	0.130	0.450	0.929	0.336	0.058	0.739	0.978
**P40**	Unknown 2	0.505	0.086	0.617	0.929	-1.332	-0.223	0.192	0.856
**P42**	3-hexenol	-0.316	-0.054	0.754	0.929	-1.450	-0.241	0.156	0.856
**P44**	1-hexanol	0.277	0.047	0.784	0.929	-0.830	-0.141	0.412	0.978

**P45**	3-methylbutyl acetate	0.061	0.010	0.952	0.967	-1.290	-0.216	0.206	0.856
**P46**	2-methylbutyl acetate	1.250	0.210	0.220	0.703	0.046	0.008	0.964	0.978
**P61**	Unknown 3	1.795	0.294	0.082	0.522	0.905	0.153	0.372	0.978
**P62**	methyl 2-hydroxy-4-methyl pentanoate	0.561	0.096	0.579	0.929	1.584	0.262	0.122	0.856
**P65**	2-methylthiolan-3-one	1.619	0.268	0.115	0.554	0.789	0.134	0.435	0.978
**P69**	1-heptanol	-0.162	-0.028	0.872	0.93	-0.391	-0.067	0.698	0.978
**P77**	ethyl hexanoate	-0.576	-0.098	0.569	0.929	-0.124	-0.021	0.902	0.978
**P80**	hexyl acetate	1.382	0.231	0.176	0.626	0.418	0.071	0.679	0.978
**P94**	2-phenylethanol	0.187	0.032	0.853	0.93	-0.132	-0.023	0.895	0.978
**P96**	Linalool	-0.432	-0.074	0.669	0.929	0.355	0.061	0.725	0.978
**P104**	octanoic acid	-0.042	-0.007	0.967	0.967	-0.054	-0.009	0.958	0.978
**P106**	ethyl octanoate	0.582	0.099	0.564	0.929	-0.398	-0.068	0.693	0.978
**P115**	2-phenethyl acetate	0.702	0.120	0.487	0.929	0.469	0.080	0.642	0.978
**P124**	nonanoic acid	1.539	0.255	0.133	0.554	-0.868	-0.147	0.392	0.978
**P129**	damascenone	0.528	0.090	0.601	0.929	-0.027	-0.005	0.978	0.978

Supplemental F

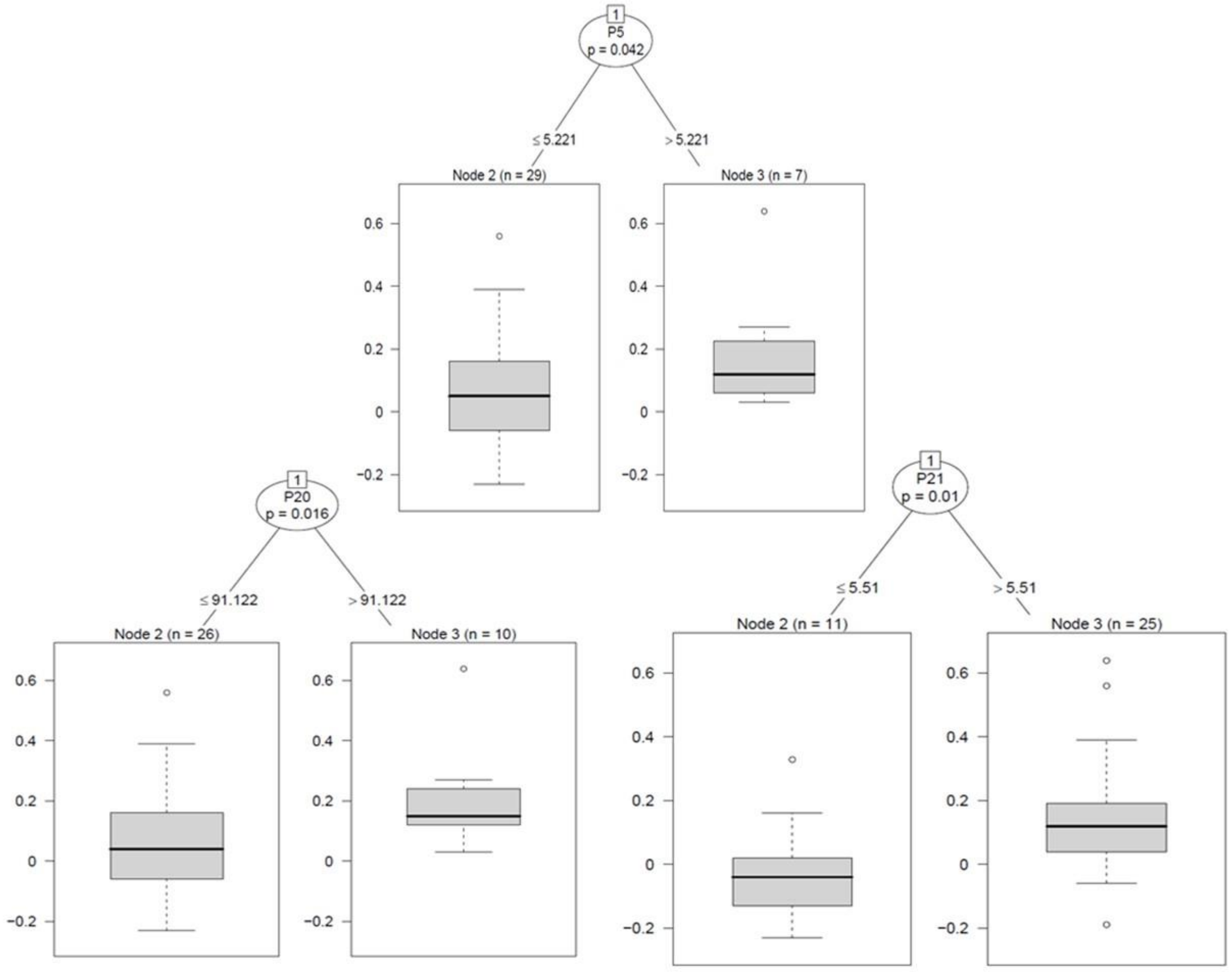

FigSF1: Conditional inference tree analysis for *Drosophila simulans* attraction (Attraction Index on y-axis) to ethyl acetate (P5), 3-methylbutanol (P20) and 2-methylbutanol (P21) in fruit. This analysis identifies if the data can be split into two groups based on the compounds concentration to create two groups that have statistically different attraction indices (the *P*-value is indicated in the top circle). The two box plots show the distribution of the attraction indices of the two groups created, with the values on the lines connecting the boxes to the top circle indicating the concentration of the compound where the split was made.

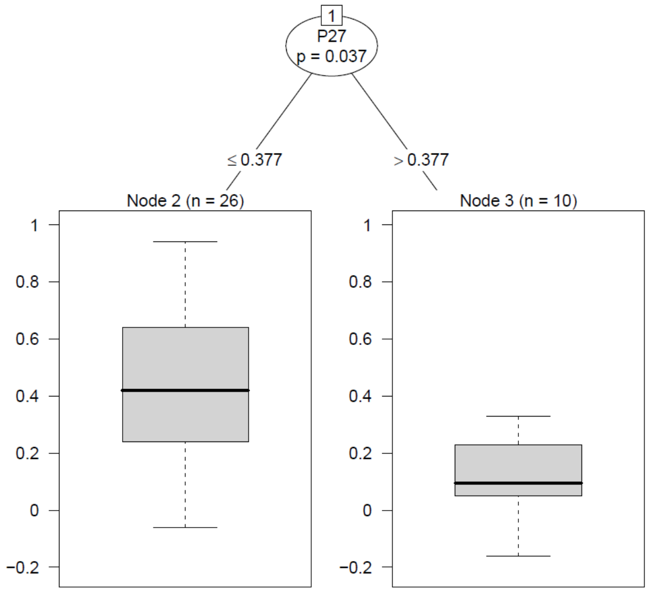

FigSF2: Conditional inference tree analysis for *Drosophila melanogaster* attraction (Attraction Index on y-axis) to 2-methylpropyl acetate (P27) in fruit. This analysis identifies if the data can be split into two groups based on the compounds concentration to create two groups that have statistically different attraction indices (the *P*-value is indicated in the top circle). The two box plots show the distribution of the attraction indices of the two groups created, with the values on the lines connecting the boxes to the top circle indicating the concentration of the compound where the split was made.
