# Supplementary material for: Are Drosophila preferences for yeasts stable or contextual?
Source: Ecol Evol. 2019 Jun 30;9(14):8075–86. doi: 10.1002/ece3.5366 (PMC6662392; doi:10.1002/ece3.5366)

Supplemental material B: Are *Drosophila*-preferences for yeasts stable or contextual?”
by Catrin S. Günther, Sarah J. Knight, Rory Jones and Matthew R. Goddard

Mass-spectral (MS) data matches against NIST –mainlibrary (2017) using MS Search 2.2 for tentative compound identification (ID) of volatiles listed in Table 1 using strawberry juice inoculated with *Saccharomyces cerevisiae* as reference.

The given retention time (RT) refers to peaks identified using gas chromatography as described in Materials and Methods. Respective MS- spectra are displayed in red (head-position) and the spectral match of suggested compound-IDs (Table 1) are displayed in blue (tail-position). Compounds are classified as ‘Unknown’ (displayed in black) when spectra could not be assigned with confidence.

Peak # 1;
RT: 1.829 min
Suggested ID: 1-propanol

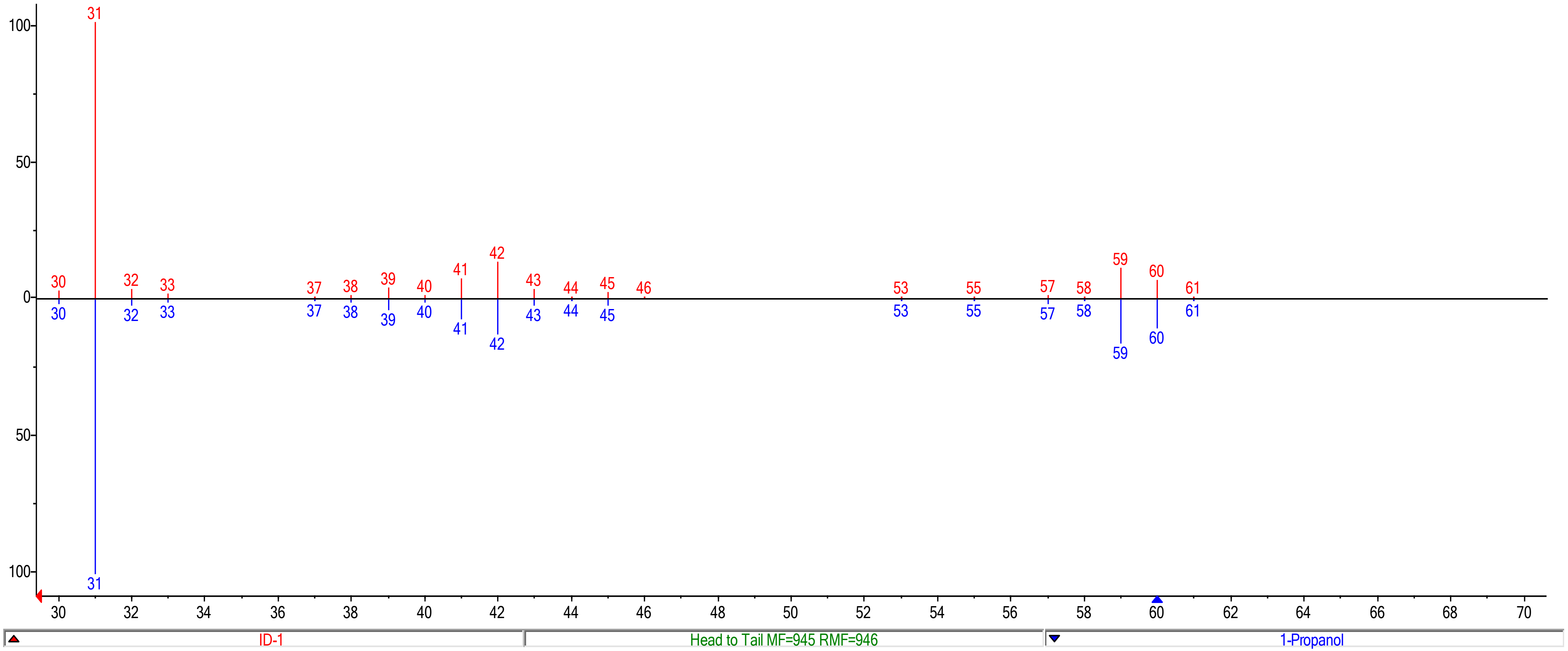

Peak # 2;
RT: 1.919 min
Suggested ID: 2,3-butanedione

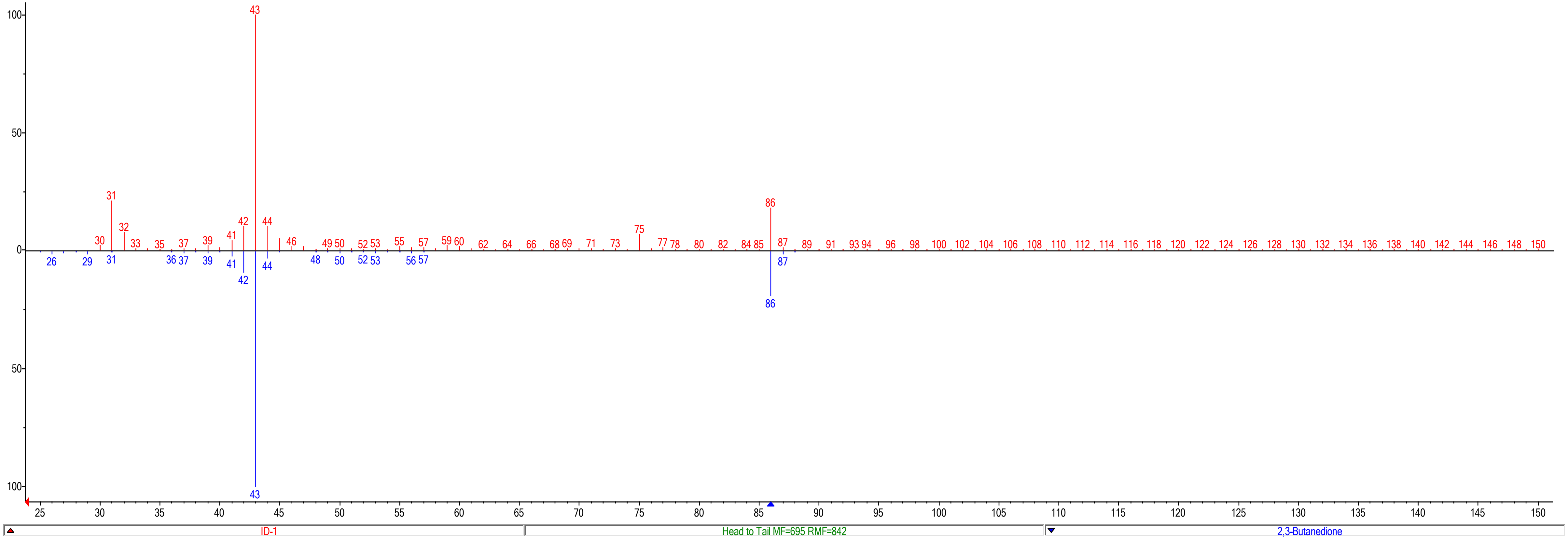

Peak # 5;
RT: 2.133 min
Suggested ID: ethyl acetate

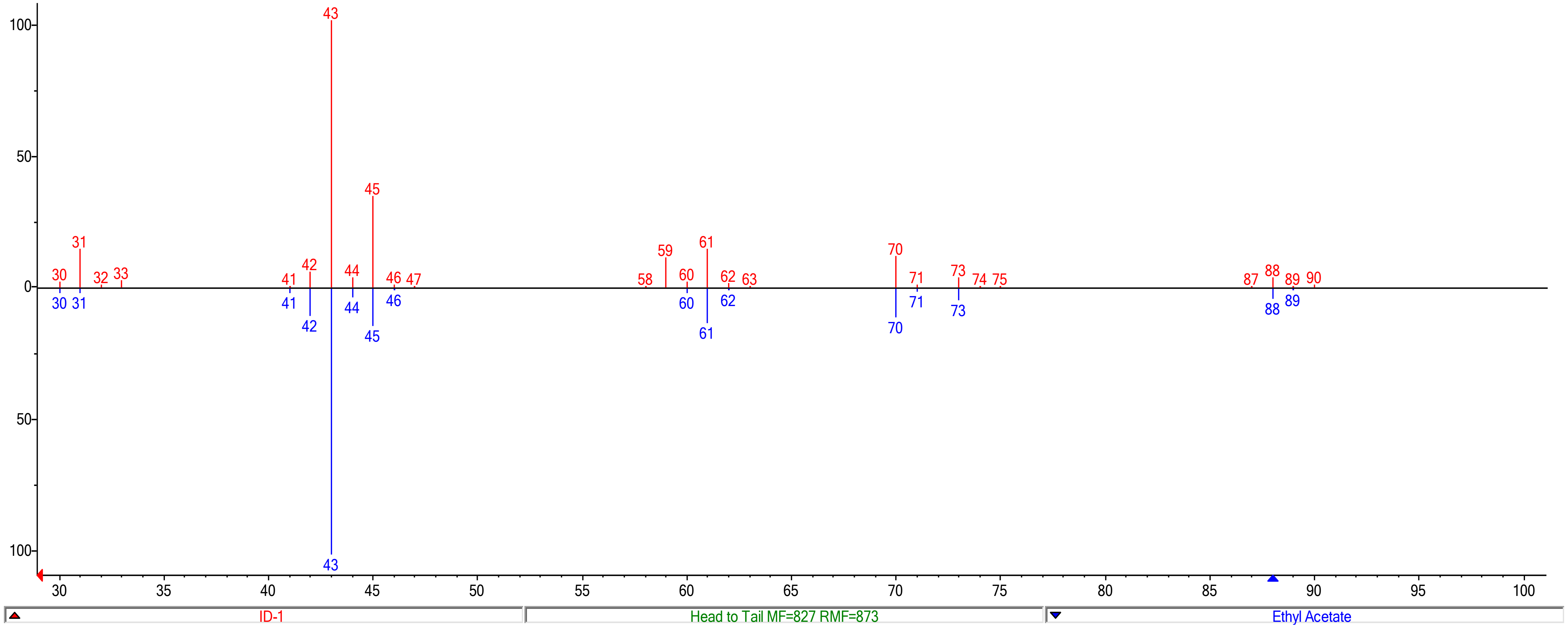

Peak # 6;
RT: 2.292 min
Suggested ID: 2-methyl 1-propanol

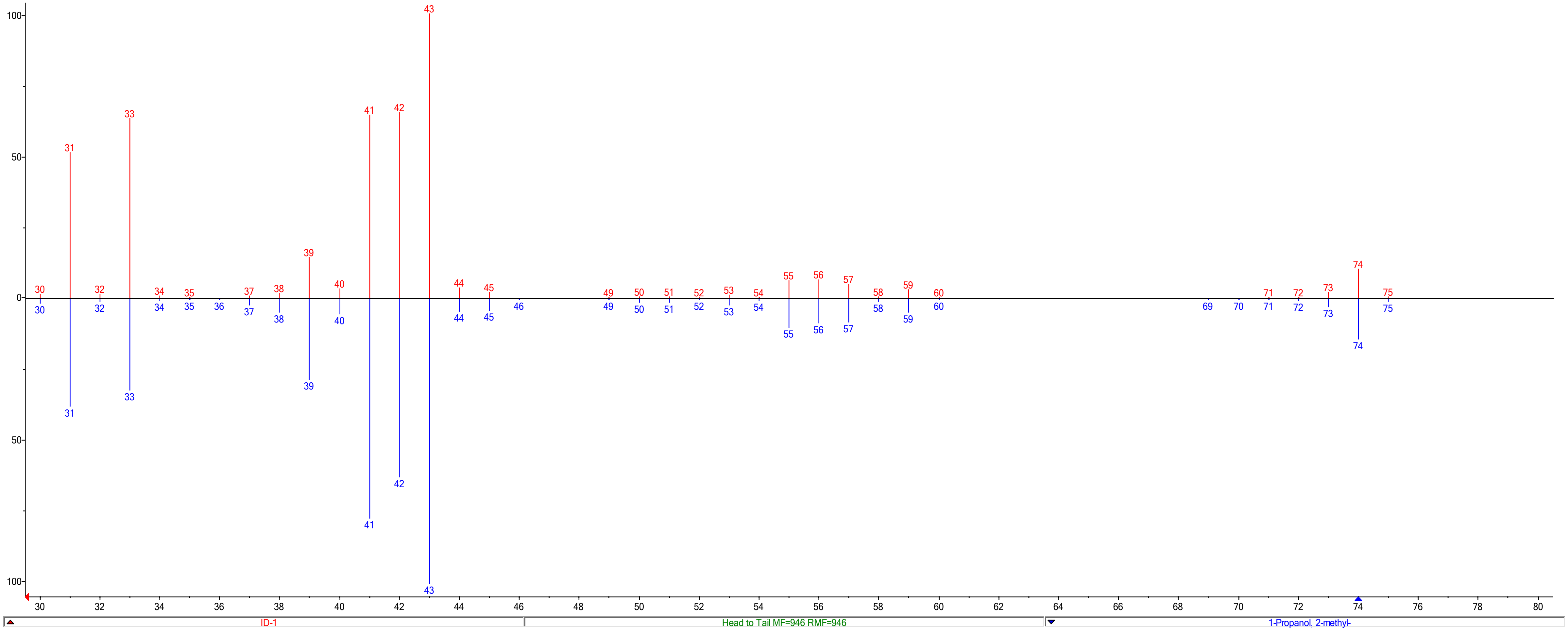

Peak # 9;
RT: 2.637 min
Suggested ID: 1-butanol

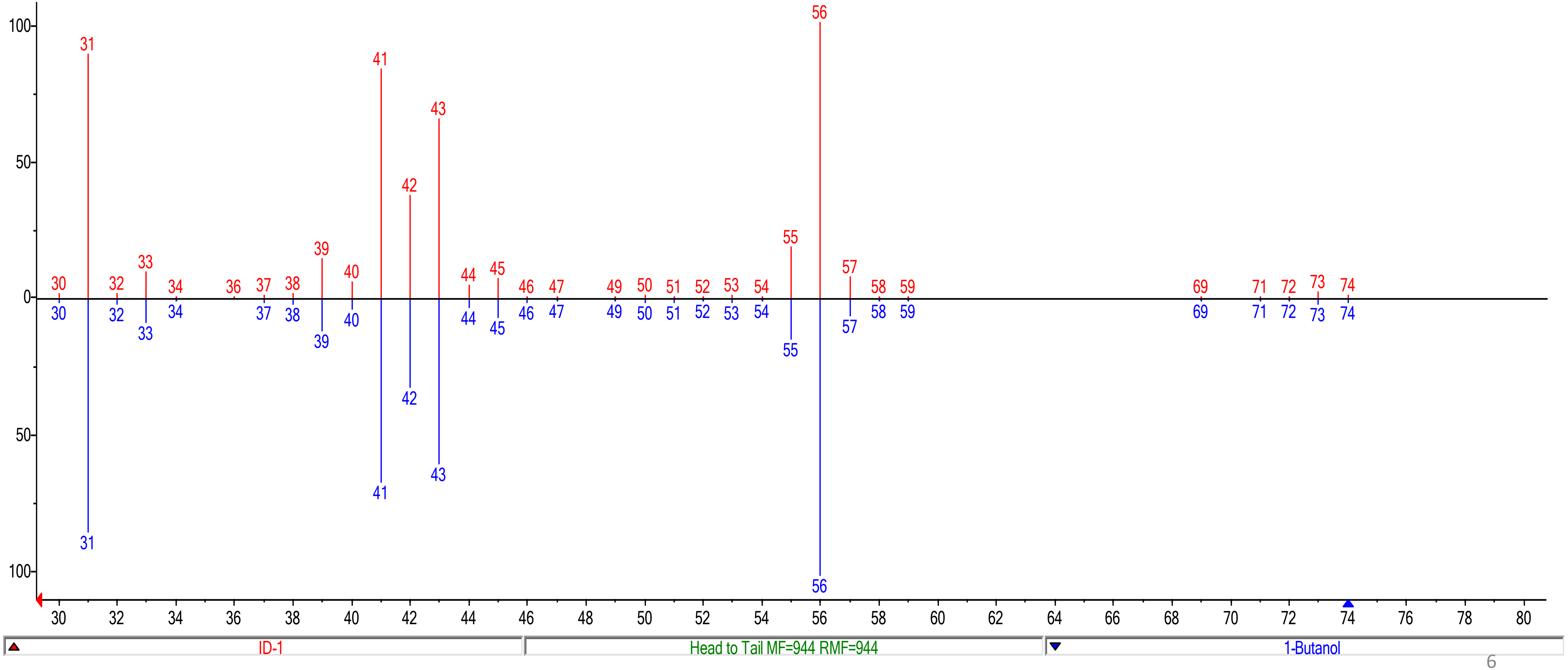

Peak # 11;
RT: 2.817 min
Suggested ID: 2-Pentanone

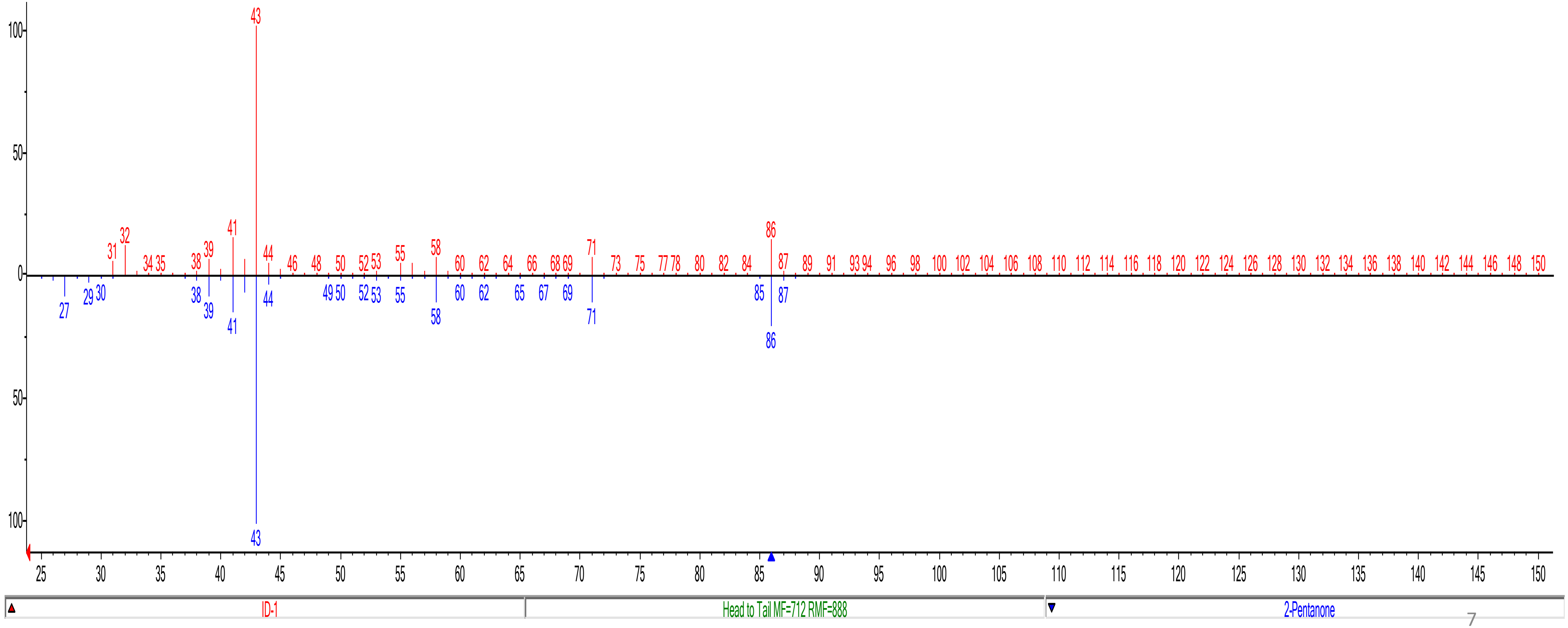

Peak # 13;

RT: 2.896 min

Suggested ID: Unknown 1

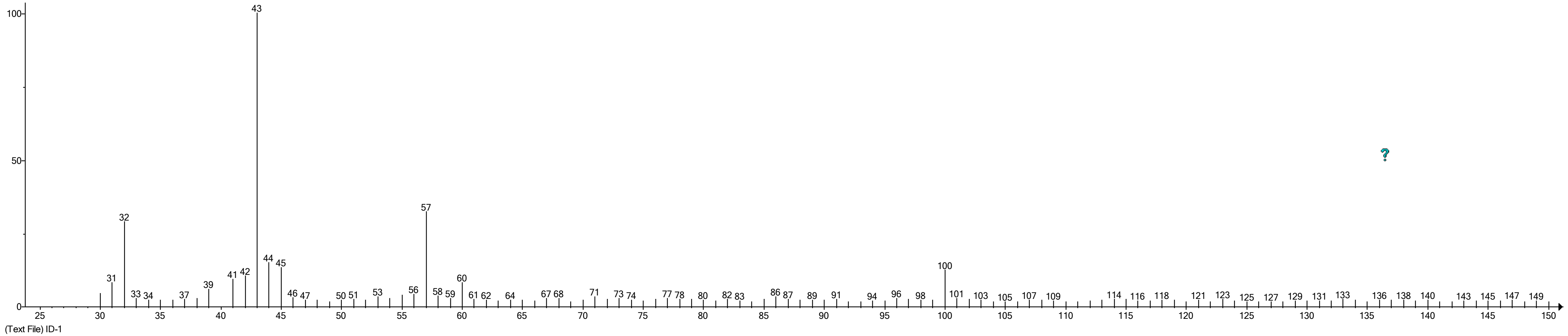

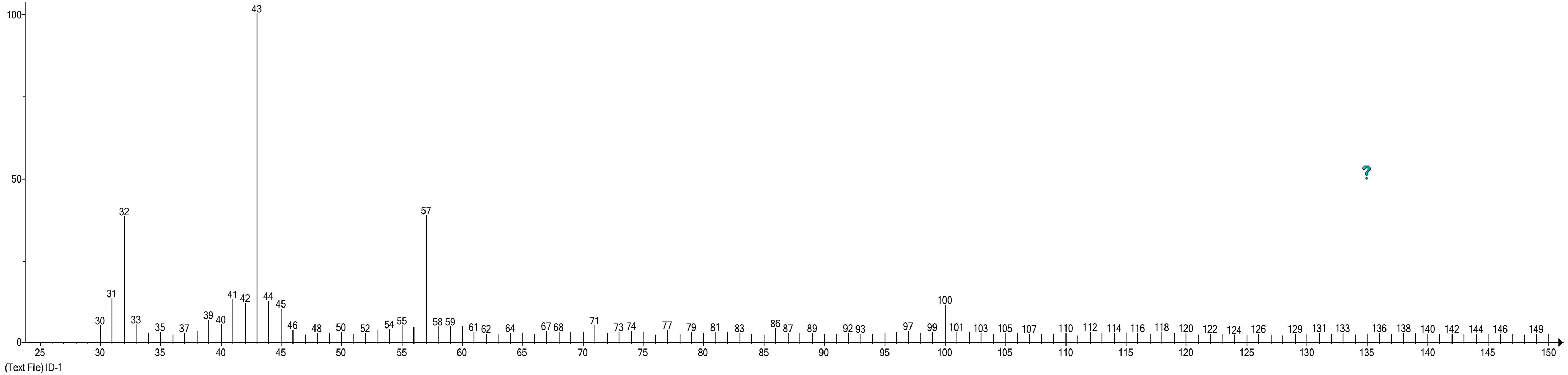

Peak # 16;
RT: 3.107 min
Suggested ID: 3-hydroxy-2-butanone

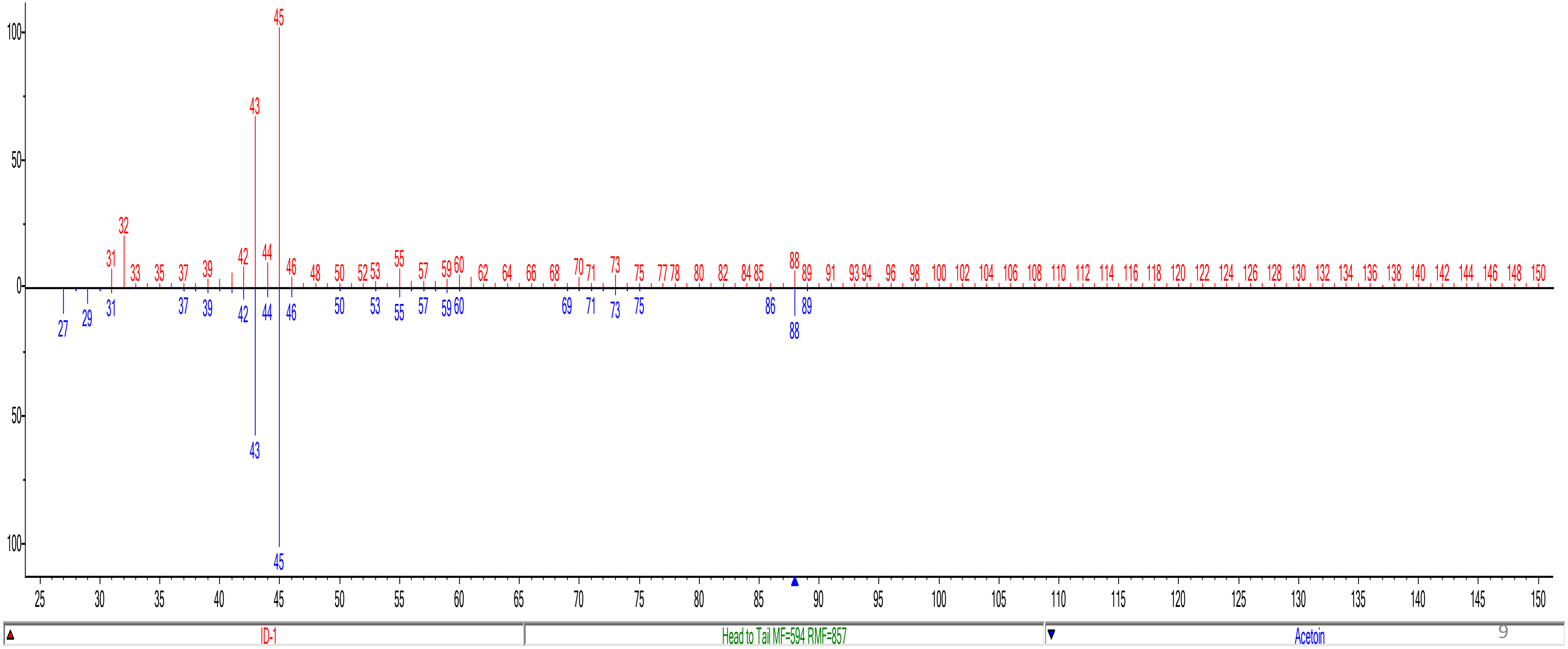

Peak # 17;
RT: 3.256 min
Suggested ID: ethyl propanoate

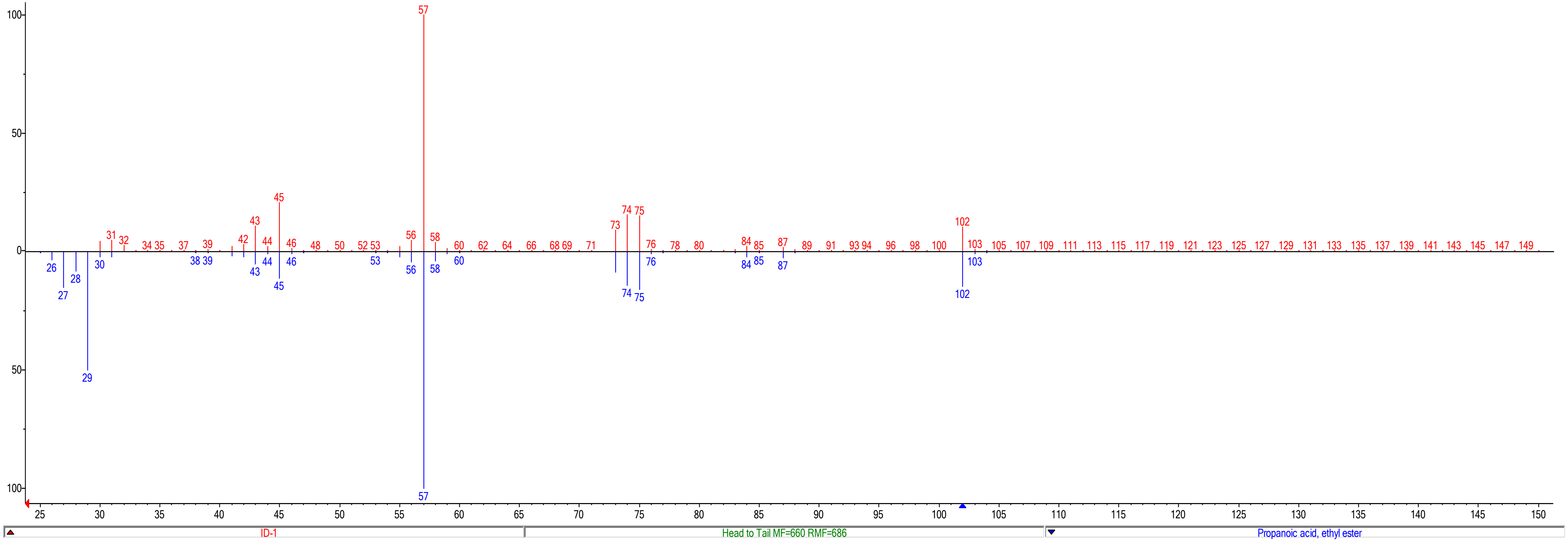

Peak # 19;
RT: 3.581 min
Suggested ID: 2,4,5-trimethyl-1,3-dioxolane (Isomer A)

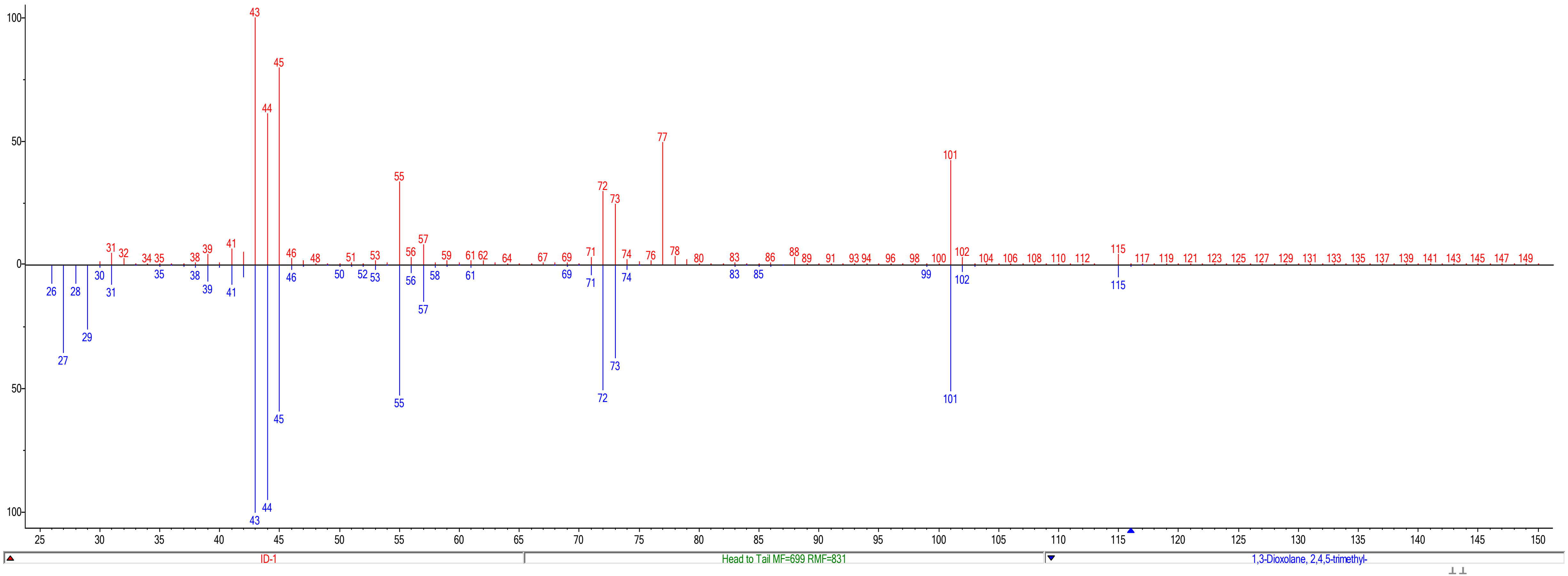

Peak # 20;

RT: 3.960 min

Suggested ID: 3-methyl butanol (Isoamyl alcohol)

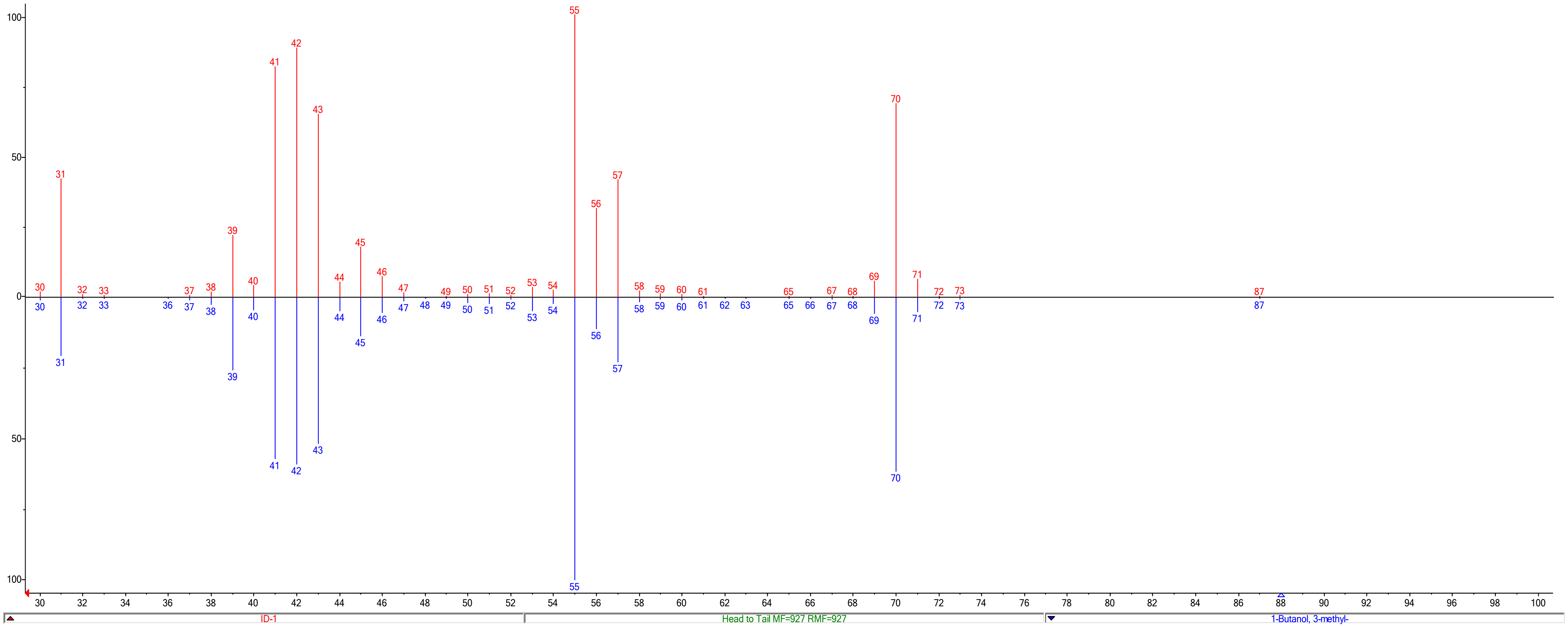

Peak # 21;
RT: 3.990 min
Suggested ID: 2-methylbutyl alcohol (amyl alcohol)

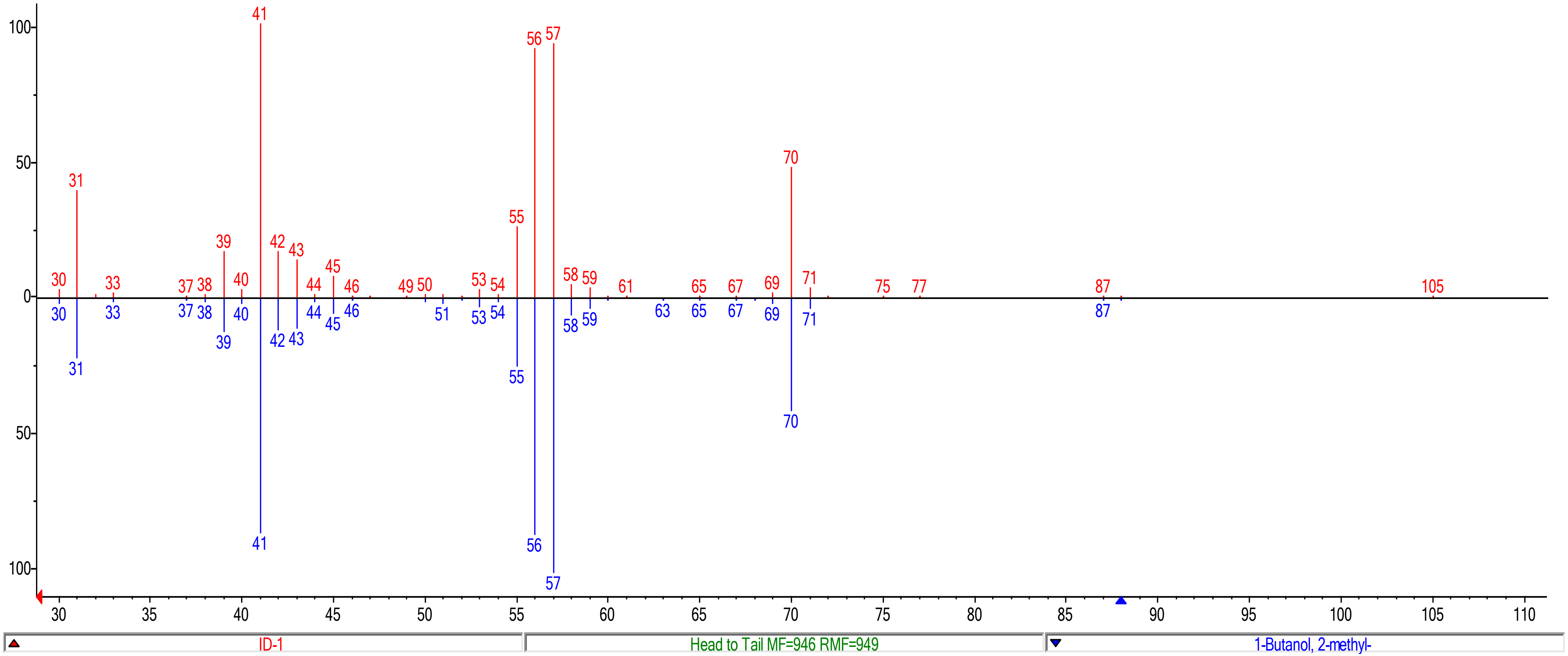

Peak # 22;
RT: 4.109 min
Suggested ID: 2,4,5-trimethyl-1,3-dioxolane (Isomer B)

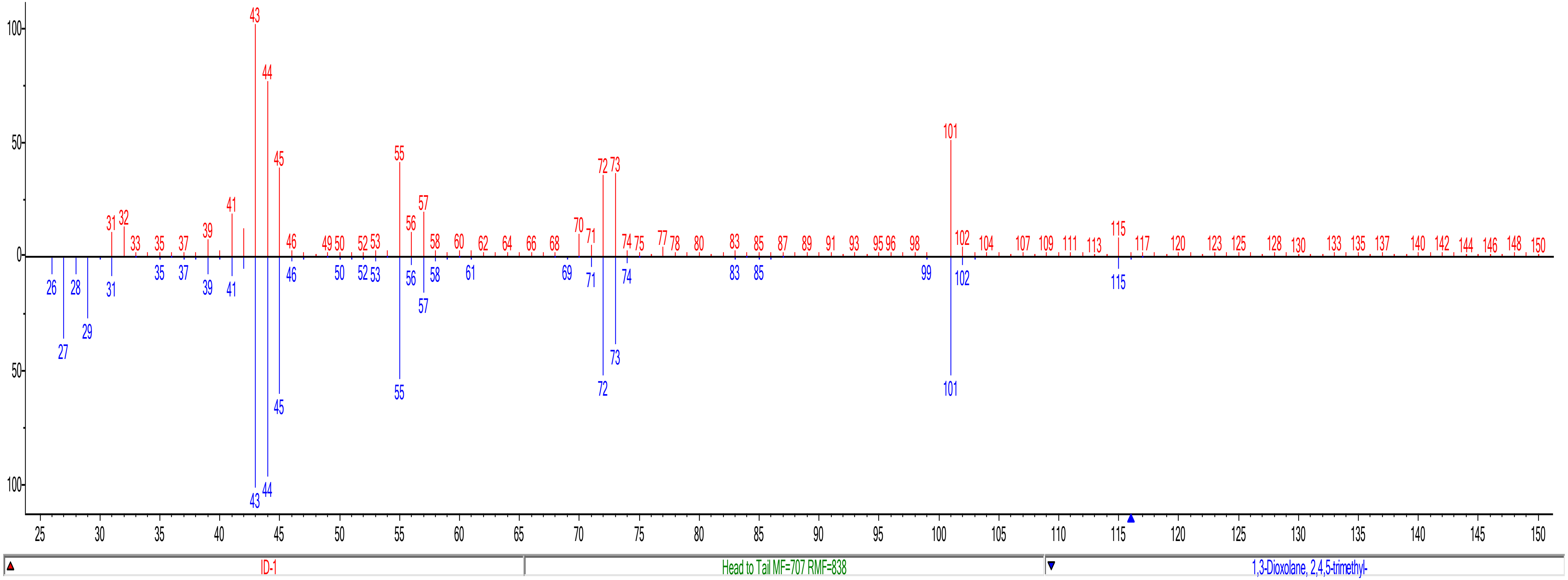

Peak # 25
RT: 4.274 min
Suggested ID: 2-methylethyl propanoate

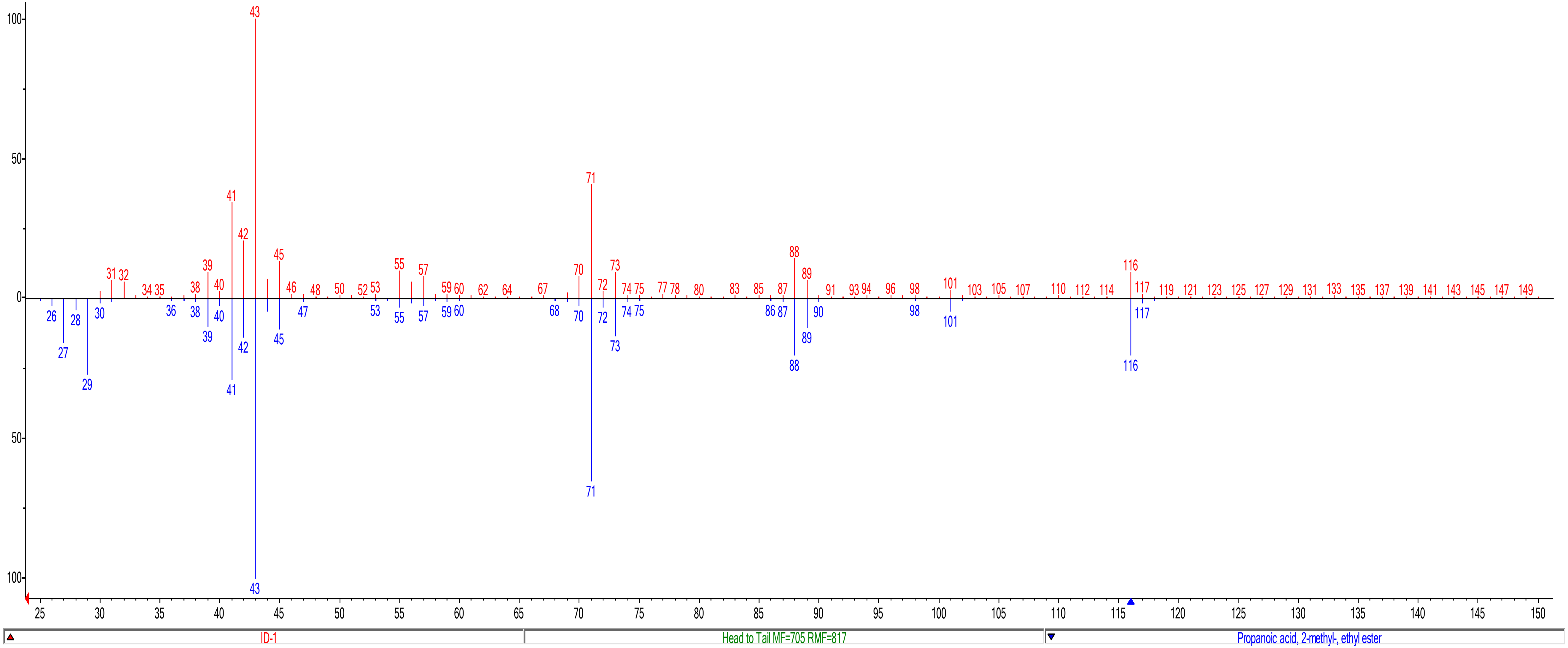

Peak # 27;
RT: 4.573 min
Suggested ID: 2-methylproyl acetate

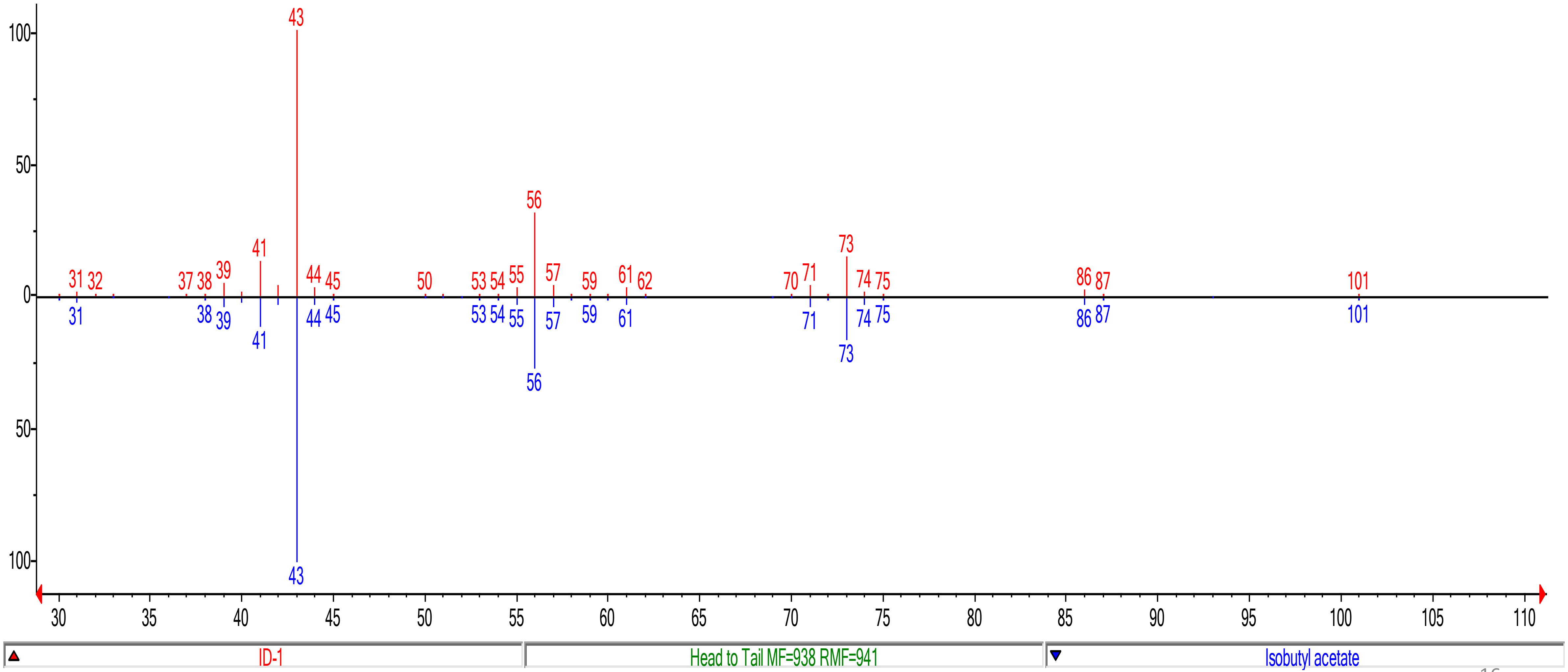

Peak # 30;
RT: 5.038 min
Suggested ID: Hexanal

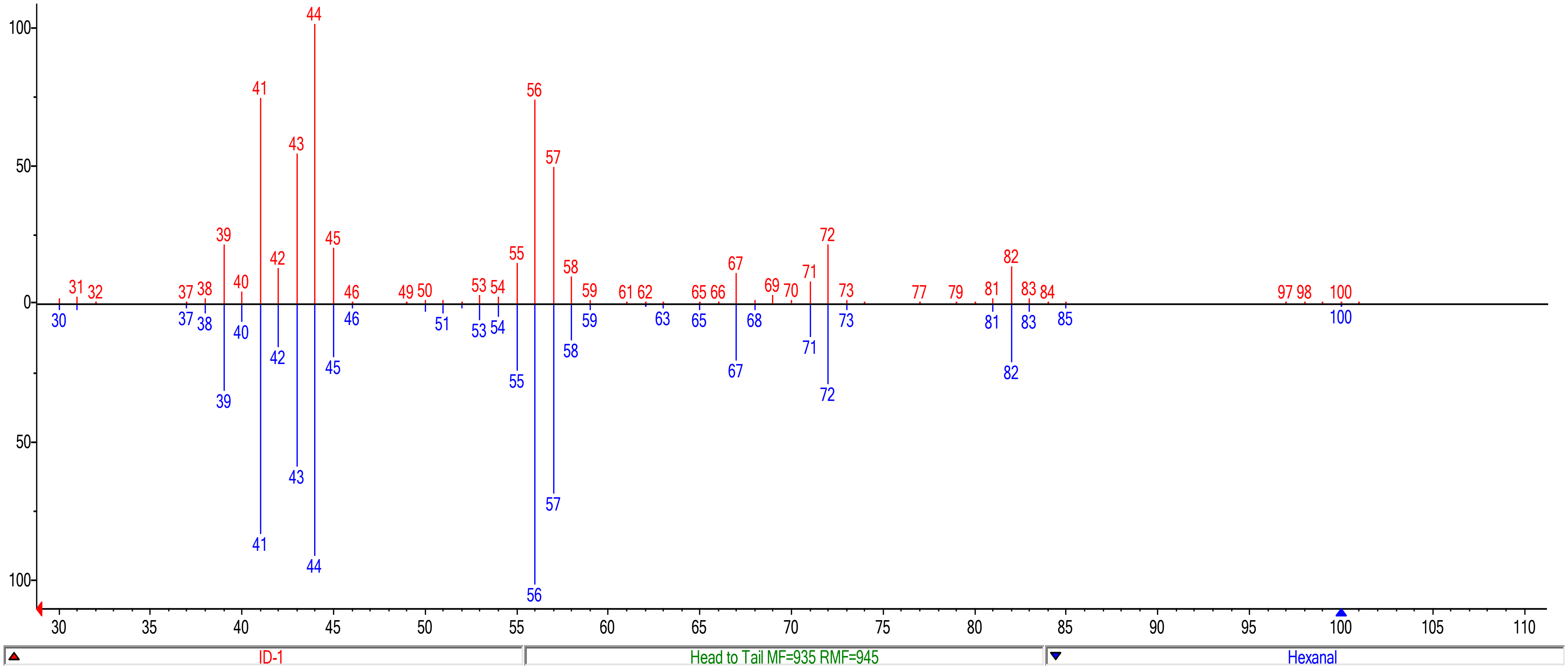

Peak # 31;

RT: 5.173 min

Suggested ID: ethyl butanoate

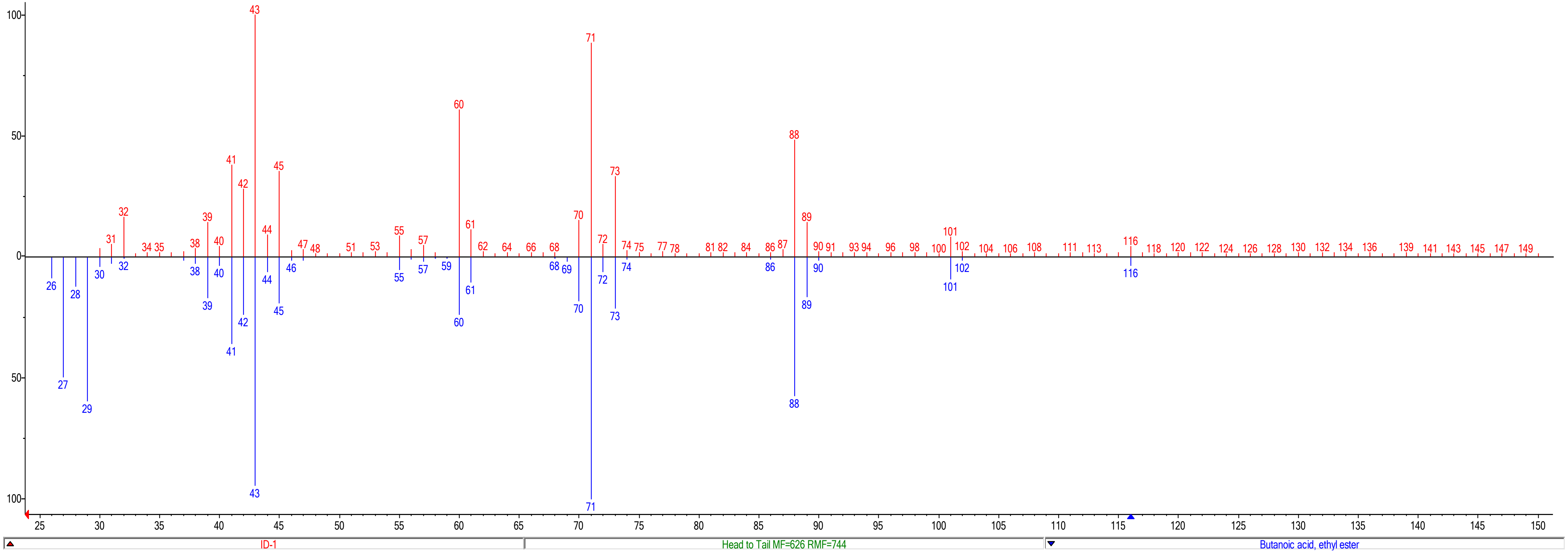

Peak # 39;
RT: 6.719 min
Suggested ID: 3-methyl-1-pentanol

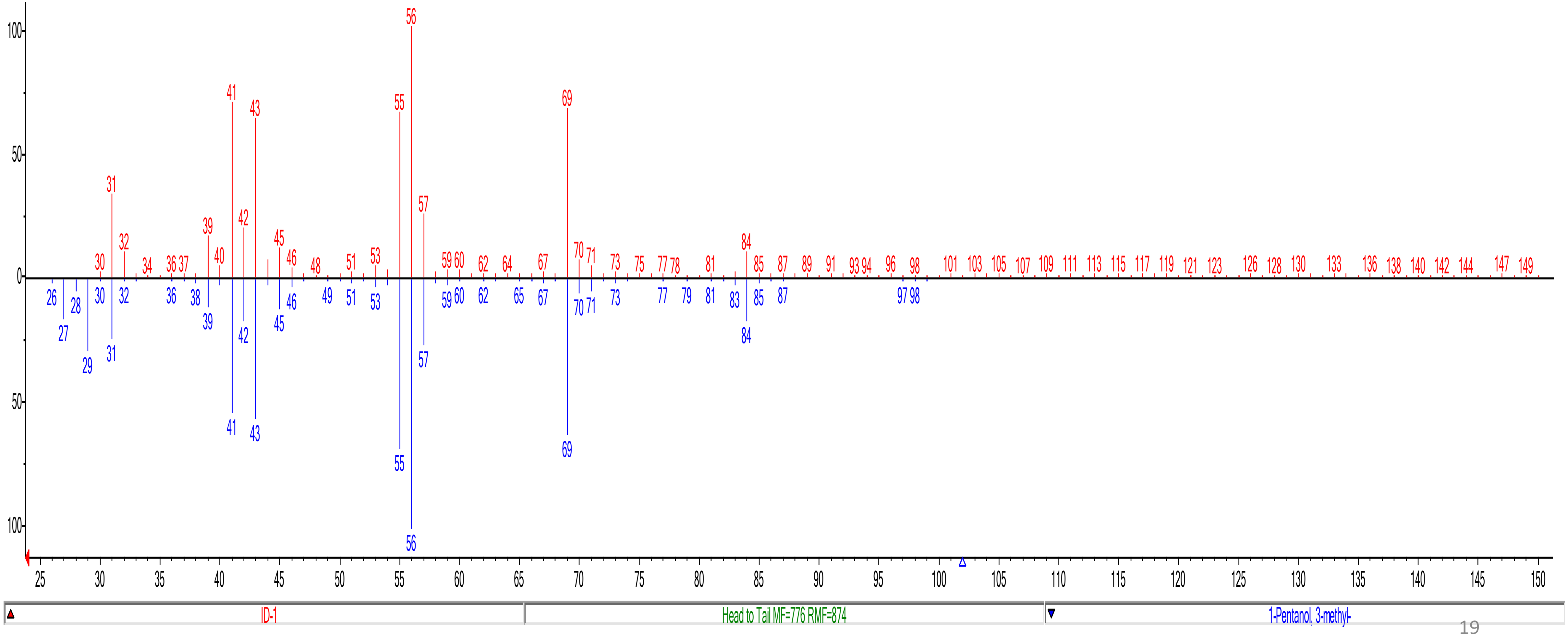

Peak # 42;
RT: 7.022 min
Suggested ID: 3-Hexen-1-ol

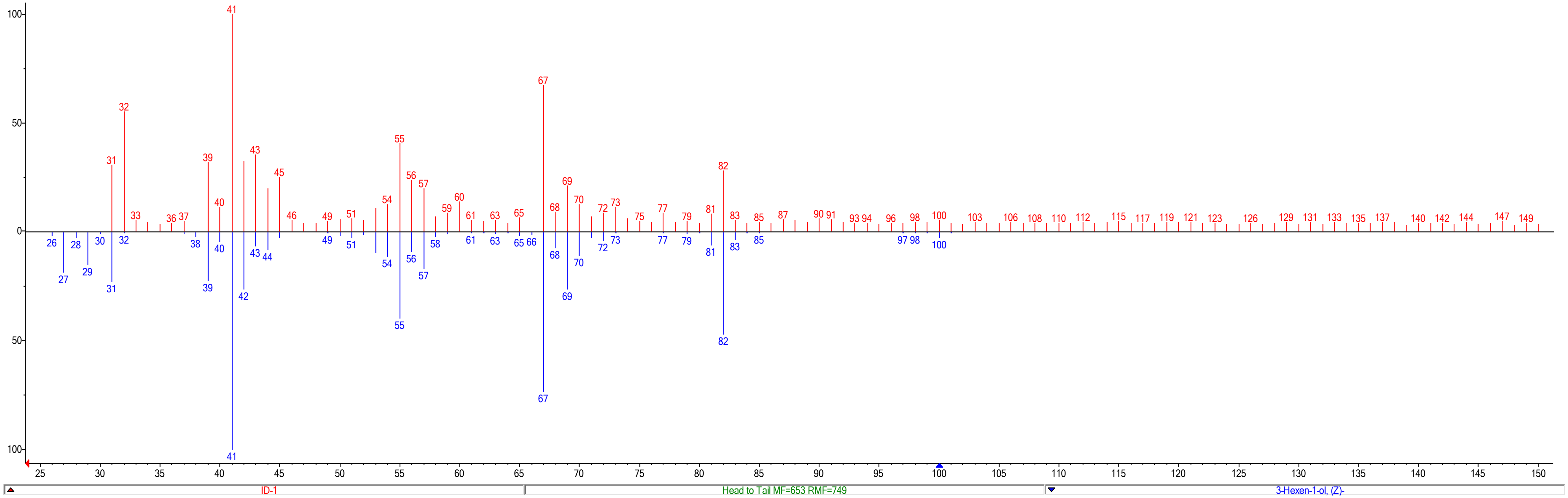

Peak # 44;
RT: 7.627 min
Suggested ID: 1-hexanol

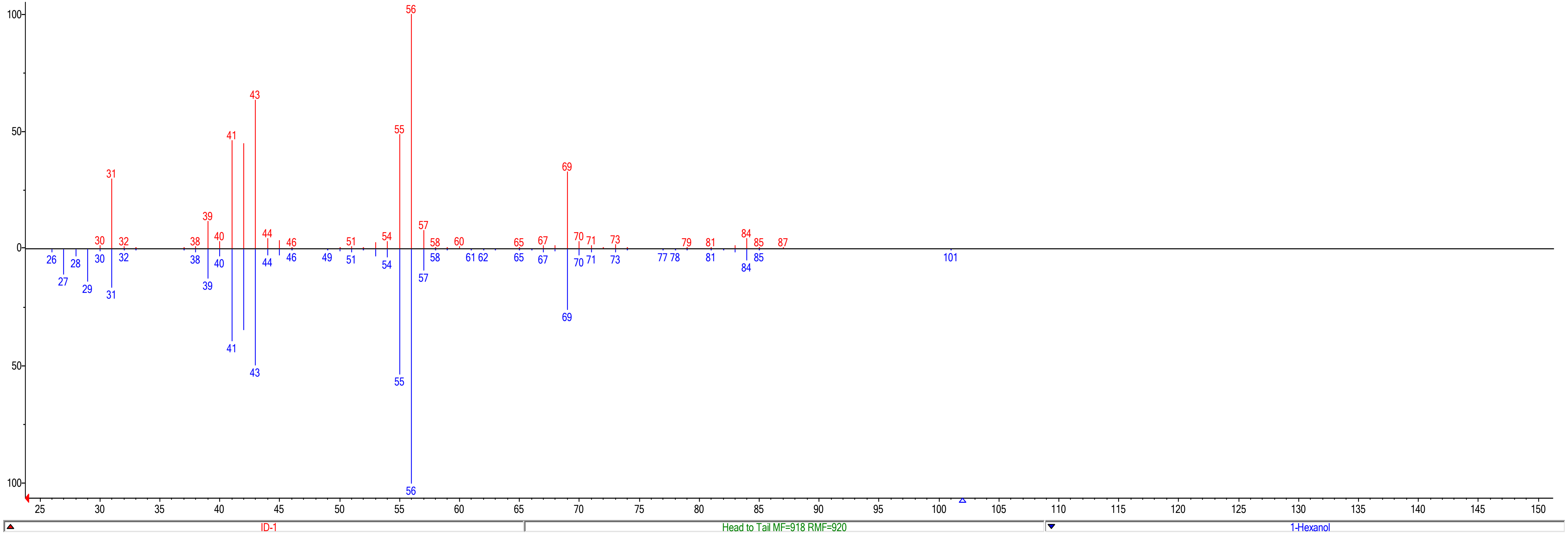

Peak # 45;
RT: 7.784 min
Suggested ID: 3-methylbutyl acetate
(Isoamyl acetate)

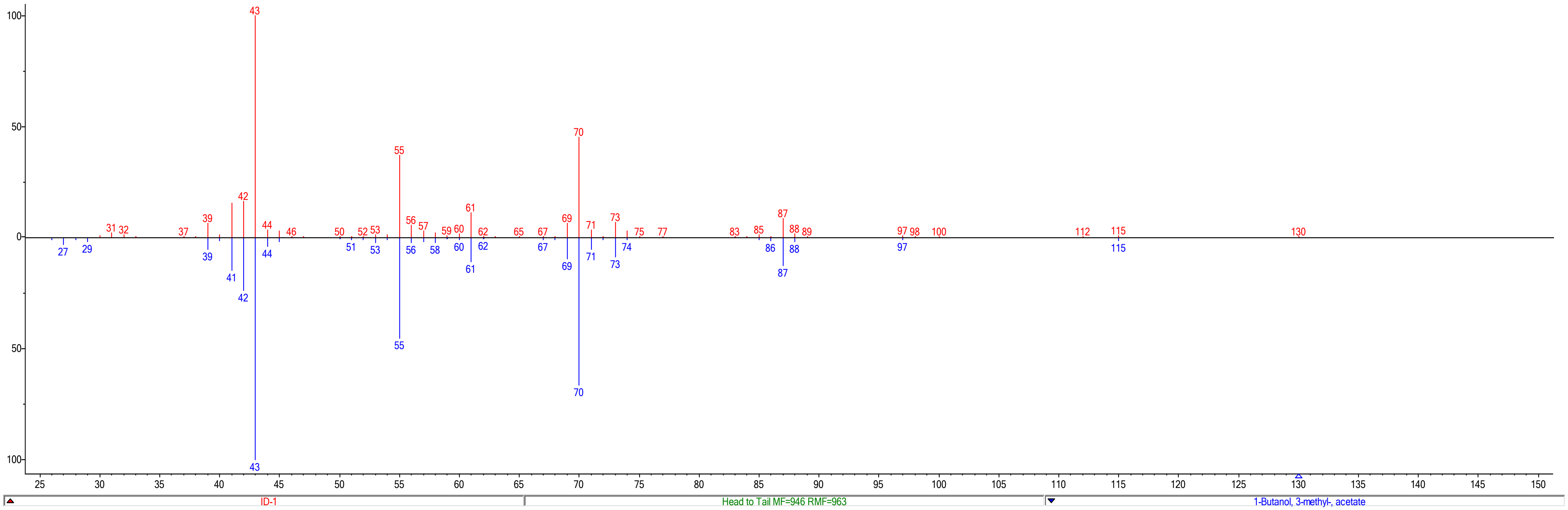

Peak # 46;
RT: 7.892 min
Suggested ID: 2-methylbutyl acetate

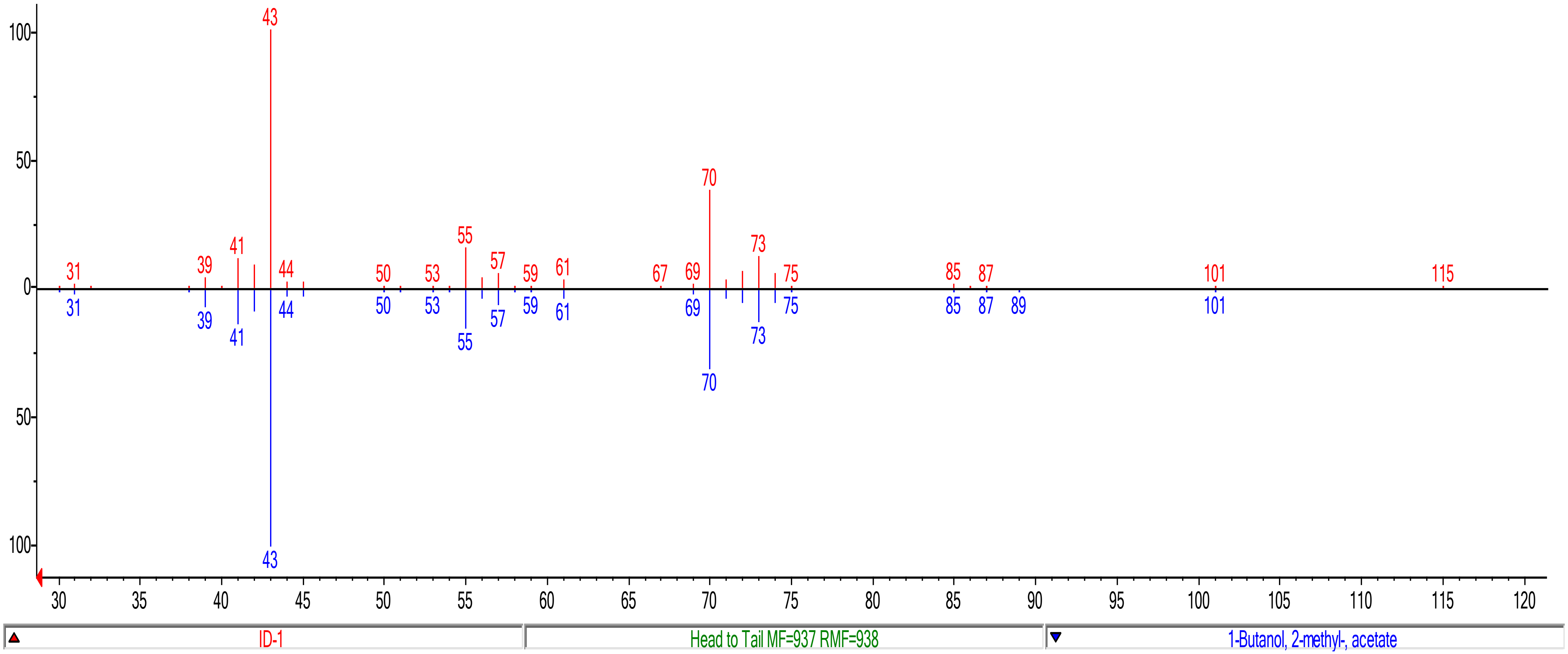

Peak # 50;
RT: 8.122 min
Suggested ID: Unknown 2

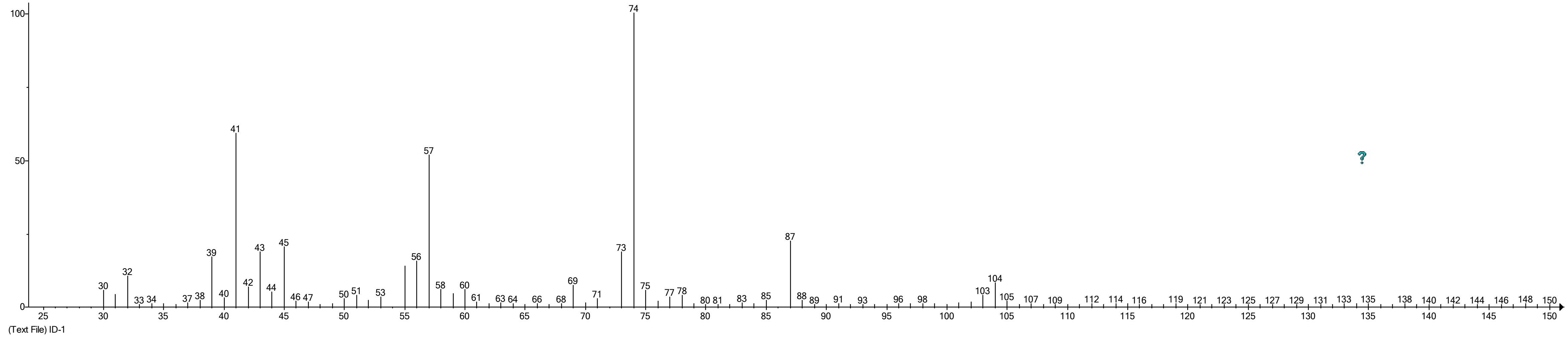

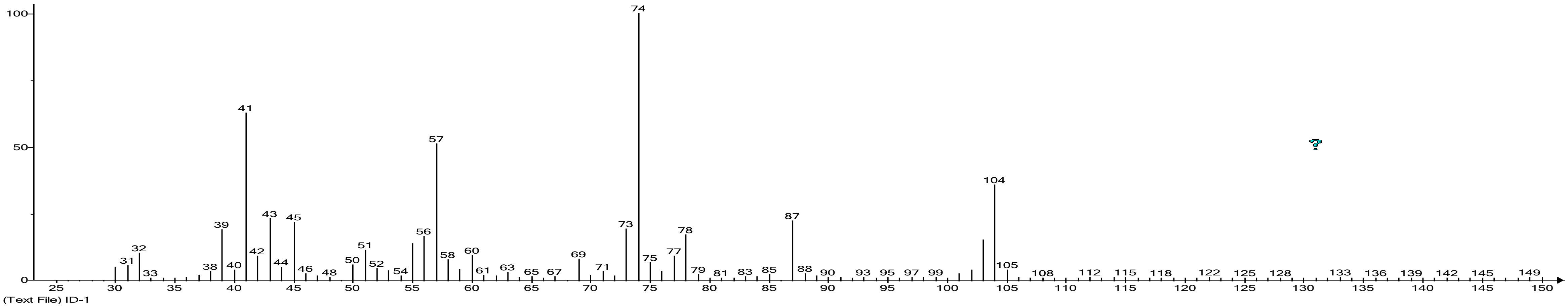

Peak # 61;
RT: 10.146 min
Suggested ID: Unknown 3

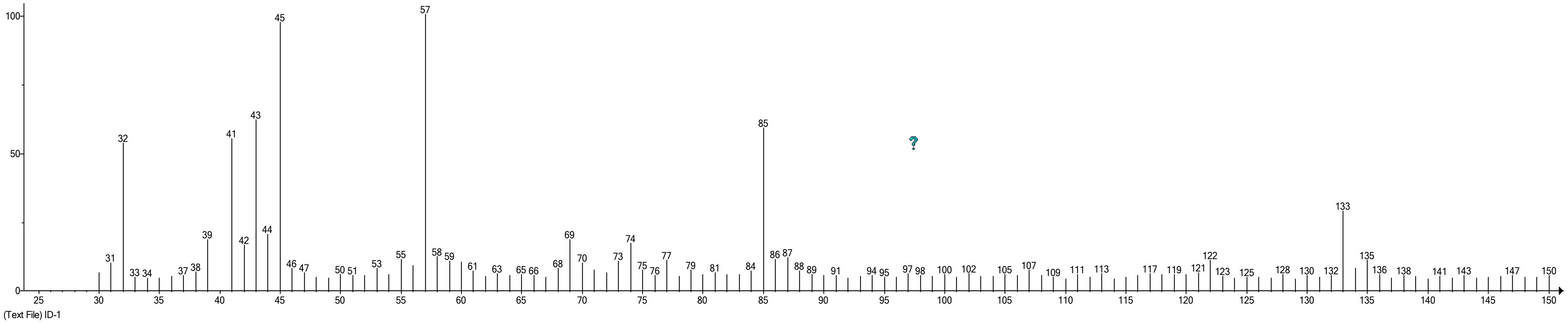

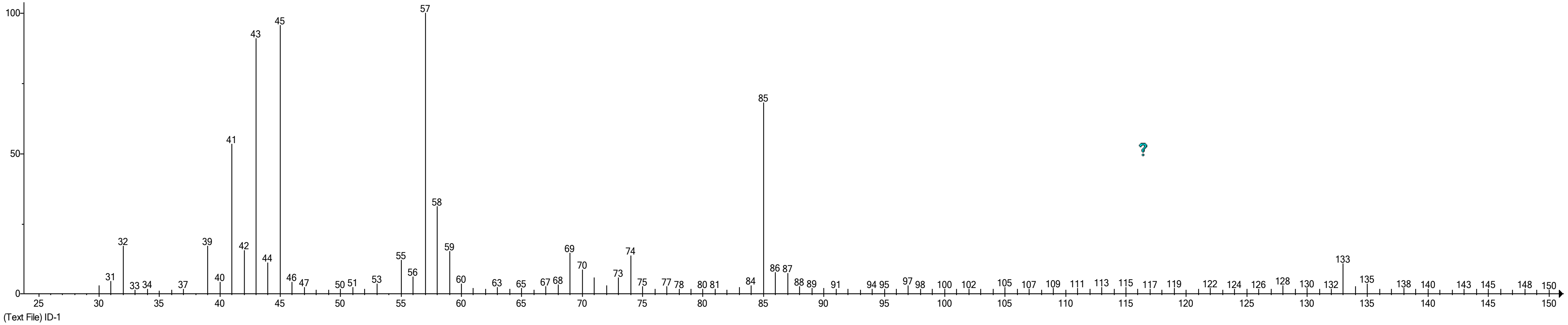

Peak # 62;

RT: 10.310 min

Suggested ID: methyl 2-hydroxy-4-methyl pentanoate

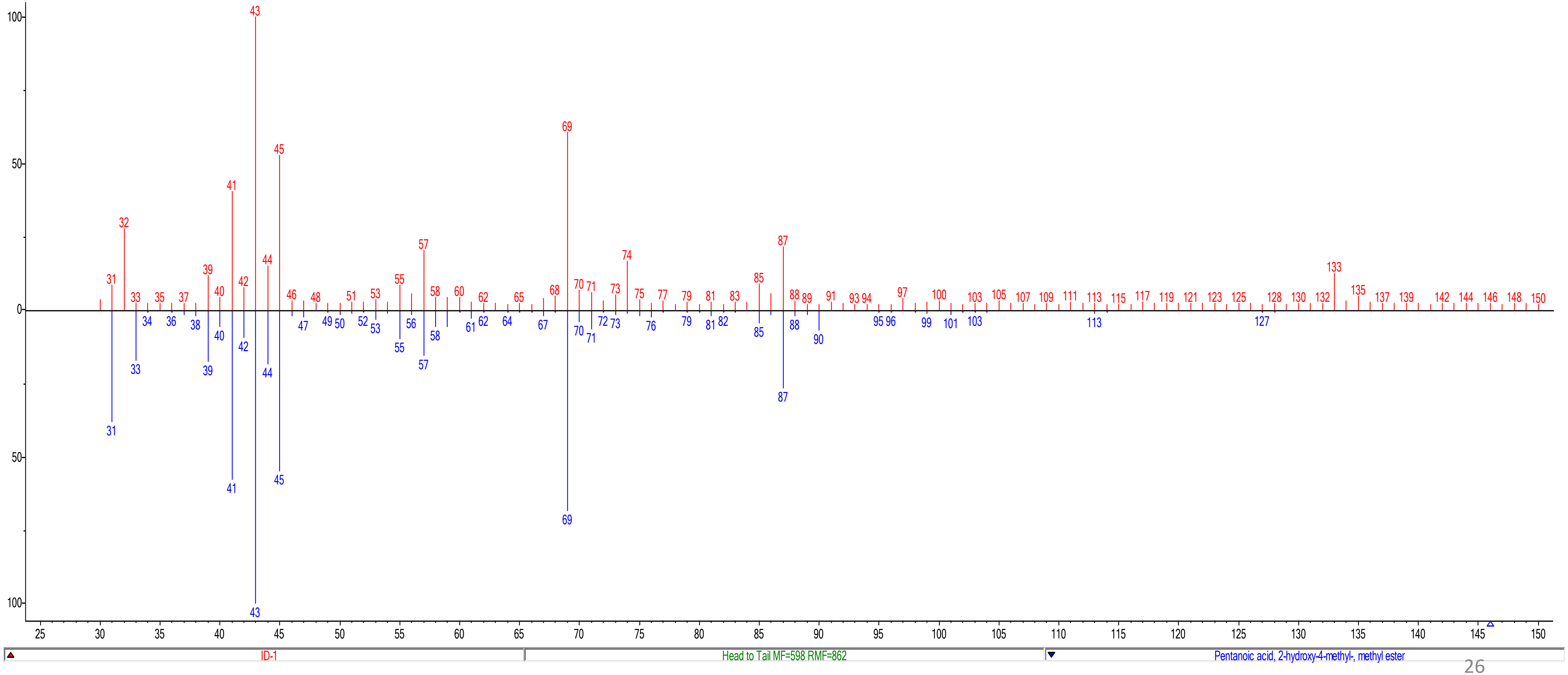

Peak # 65;
RT: 11.256 min
Suggested ID: dihydro-2-methyl-3(2H)-thiophenone
(blackberry thiophenone)

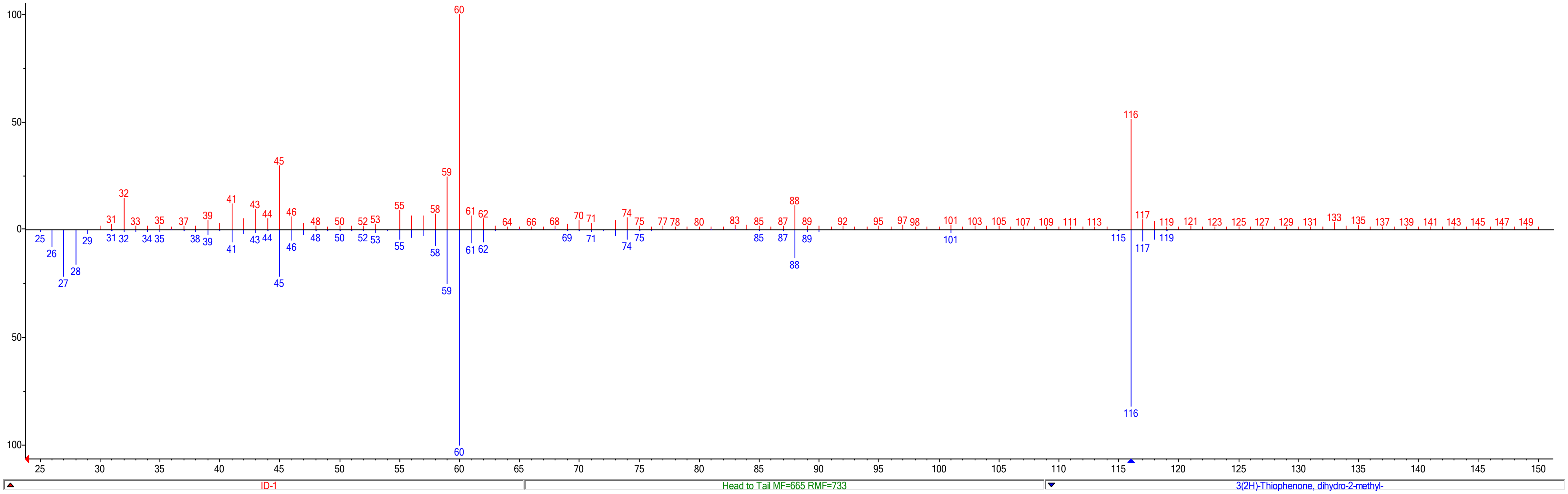

Peak # 69;
RT: 11.830 min
Suggested ID: 1-heptanol

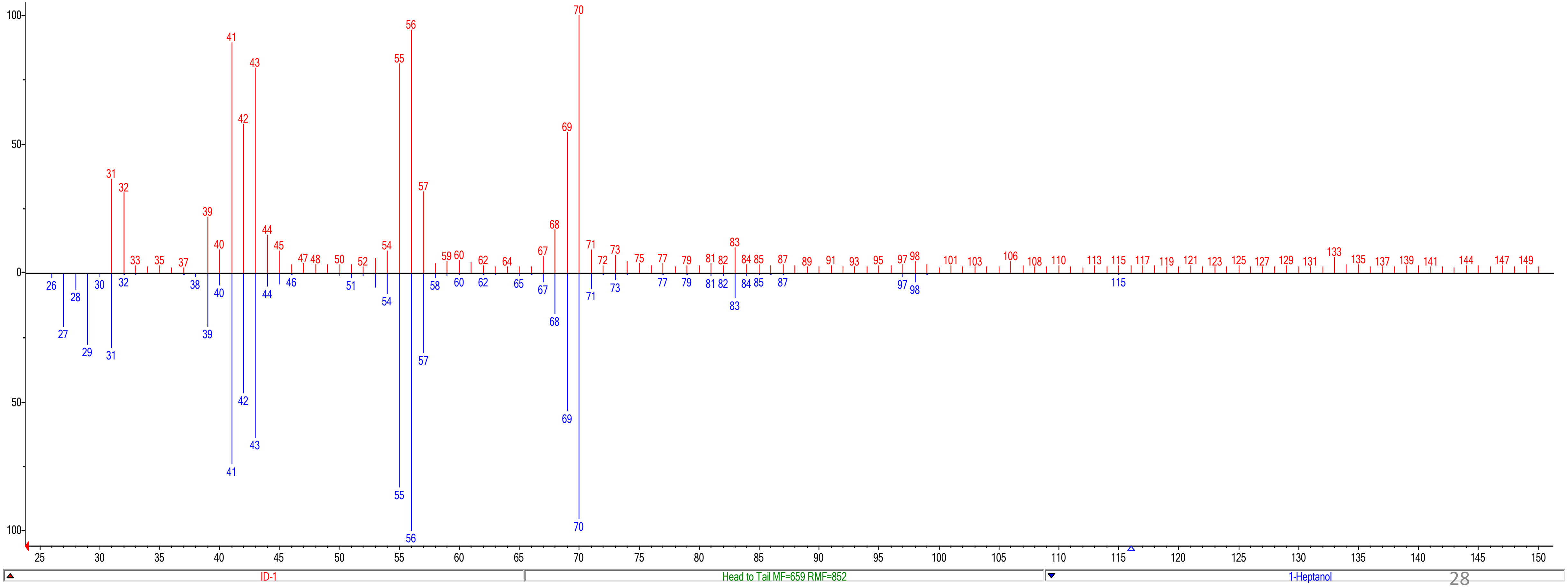

Peak # 77;
RT: 13.107 min
Suggested ID: ethyl hexanoate

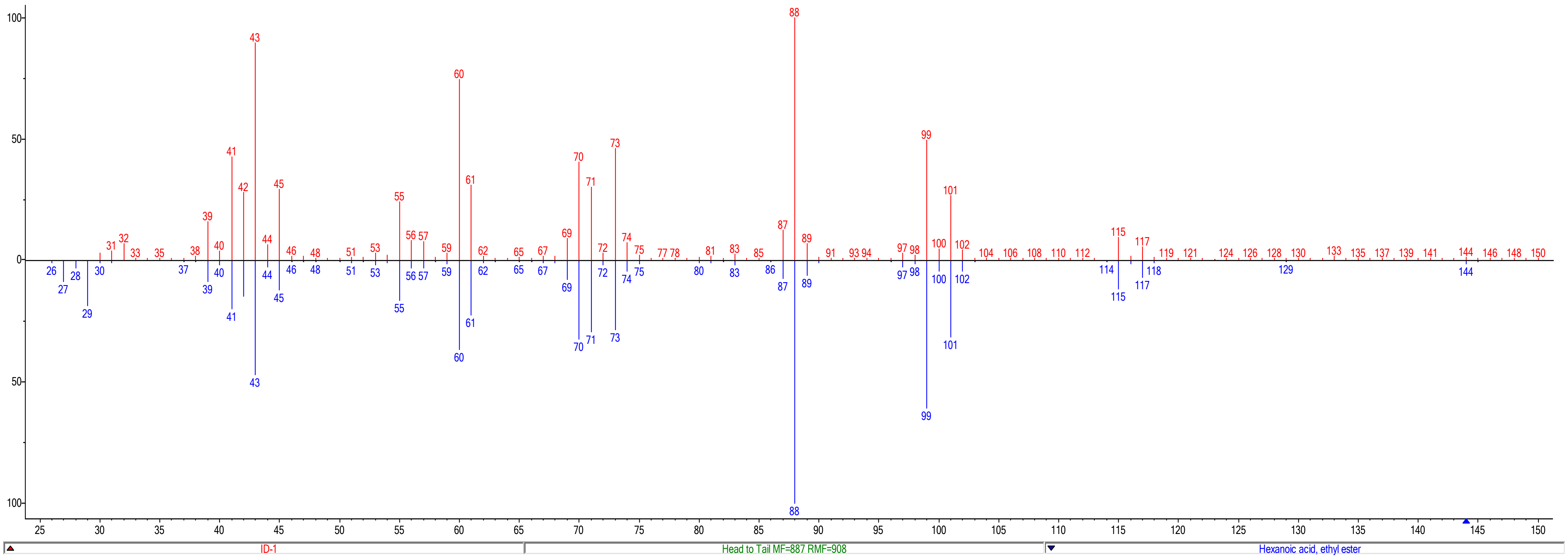

Peak # 80;
RT: 13.747 min
Suggested ID: hexyl acetate

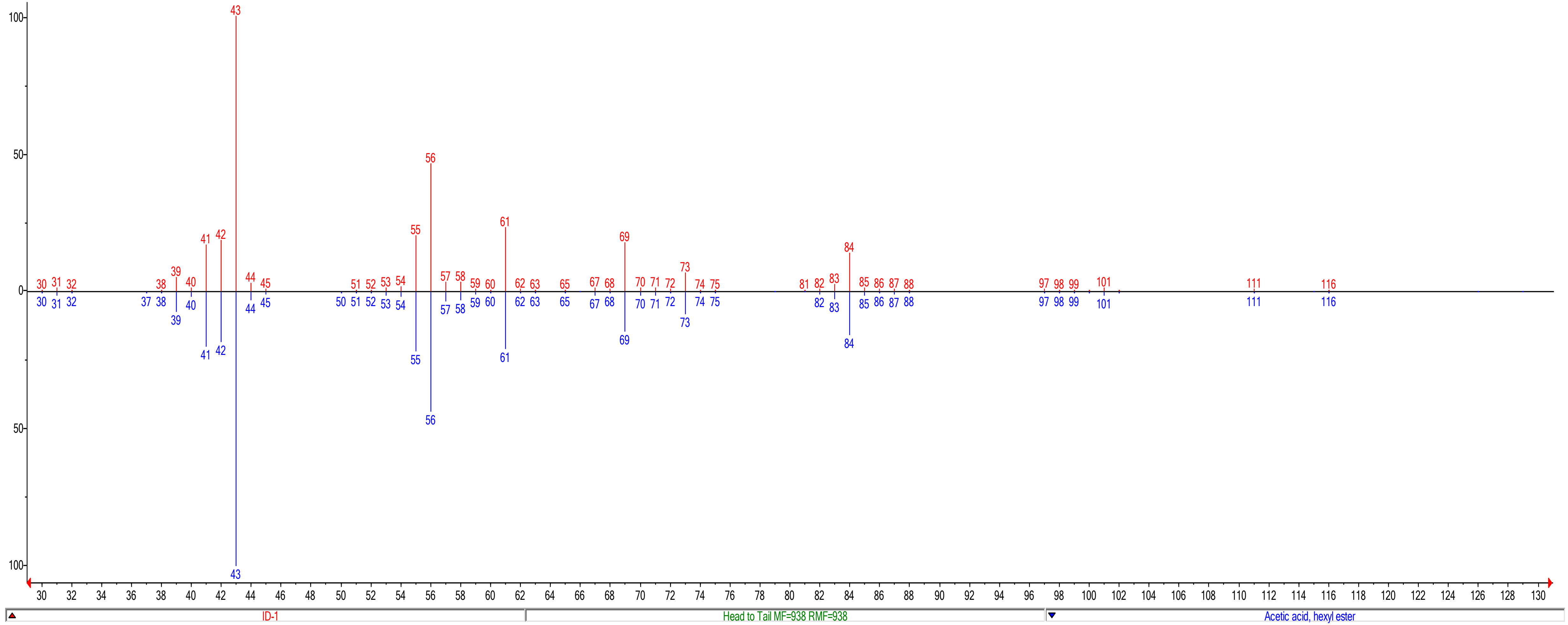

Peak # 94;
RT: 17.760 min
Suggested ID: 2-phenylethanol

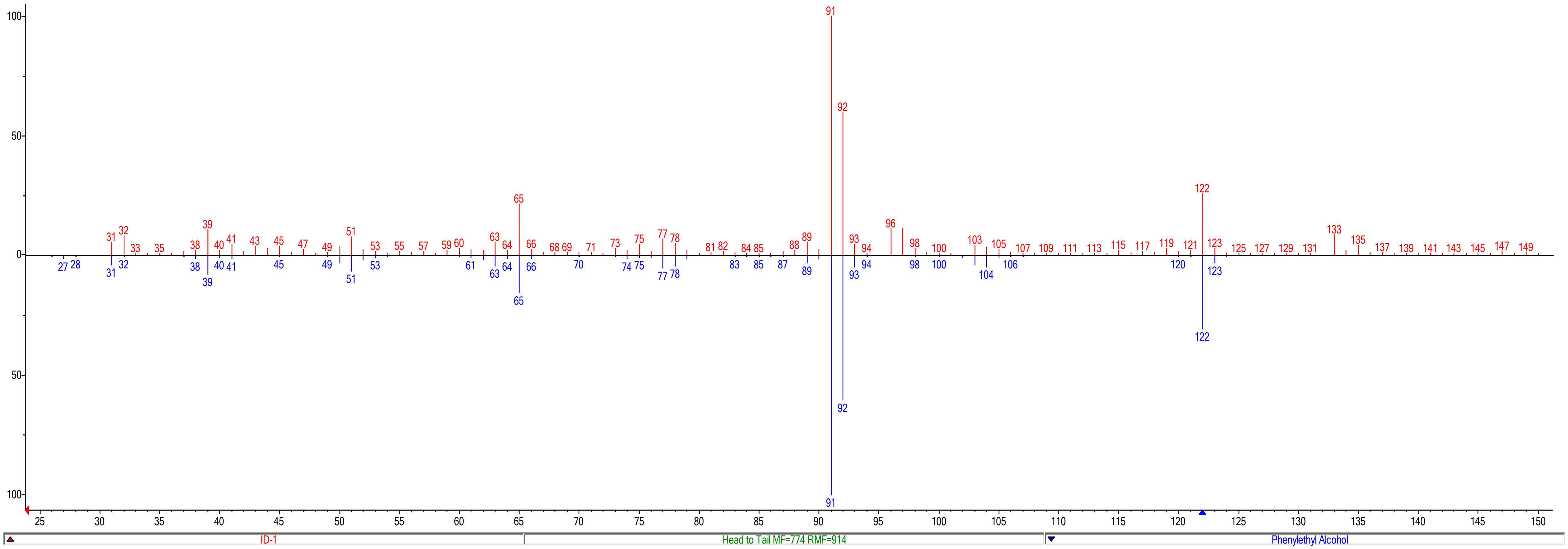

Peak # 96;
RT: 17.903 min
Suggested ID: Linalool

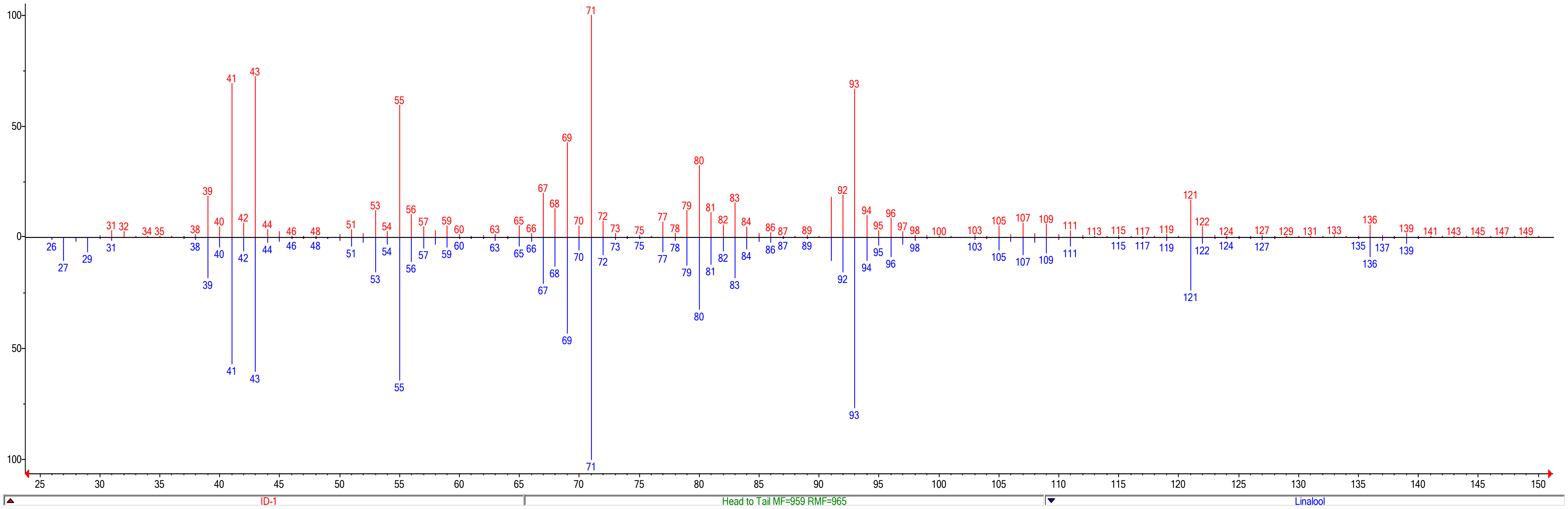

Peak # 104;
RT: 21.953 min
Suggested ID: Octanoic acid

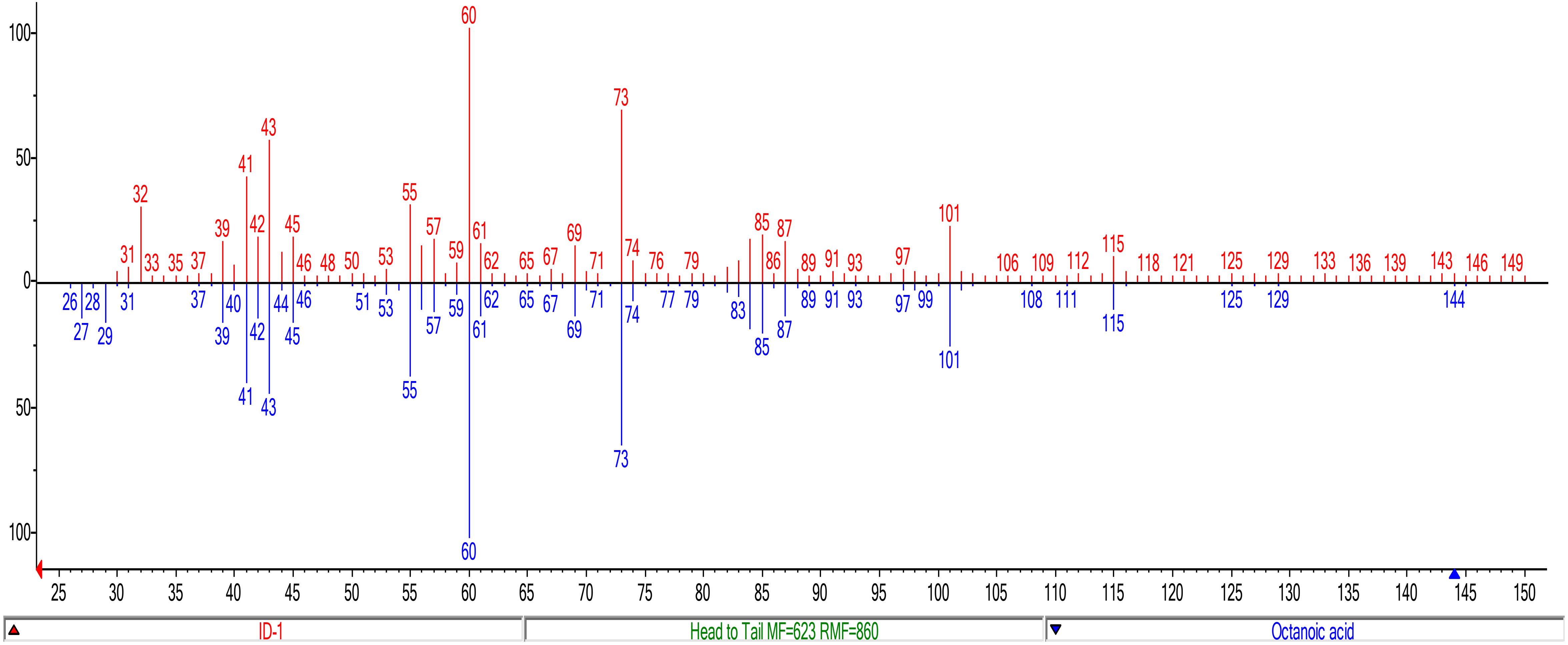

Peak # 106;
RT: 22.561 min
Suggested ID: ethyl octanoate

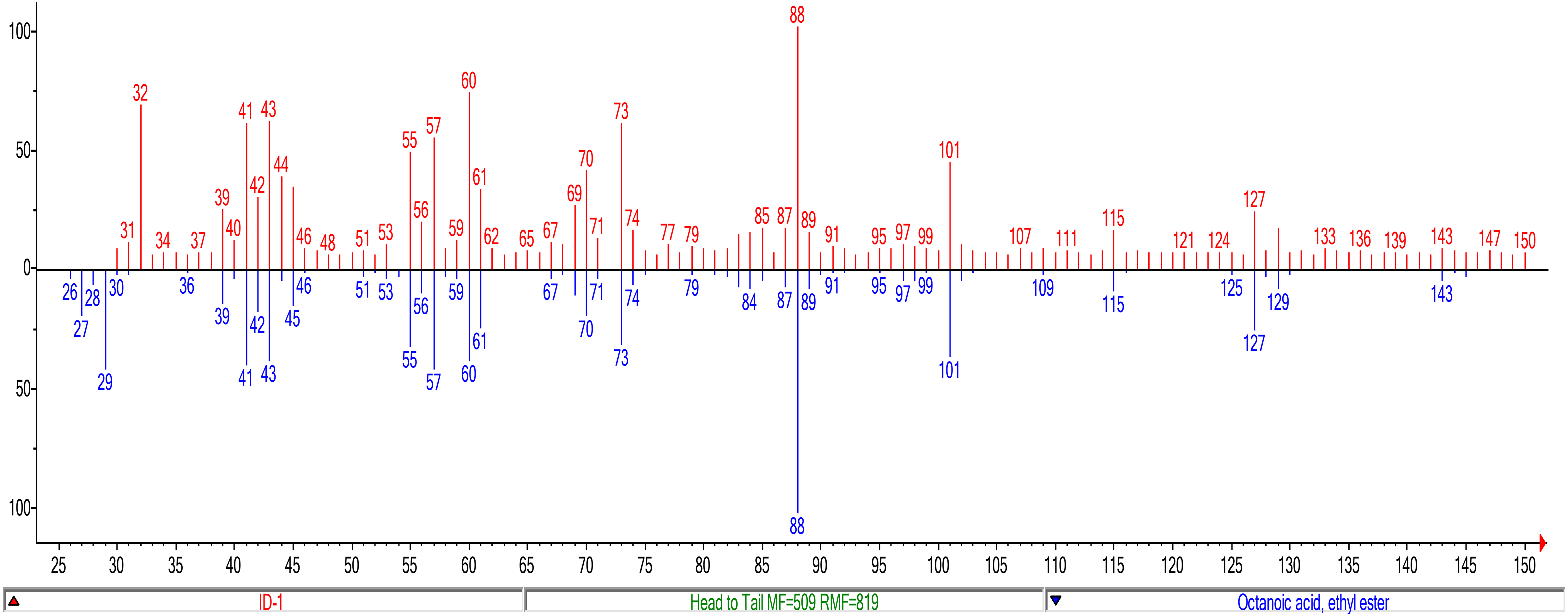

Peak # 115;
RT: 24.266 min
Suggested ID: 2-phenethyl acetate

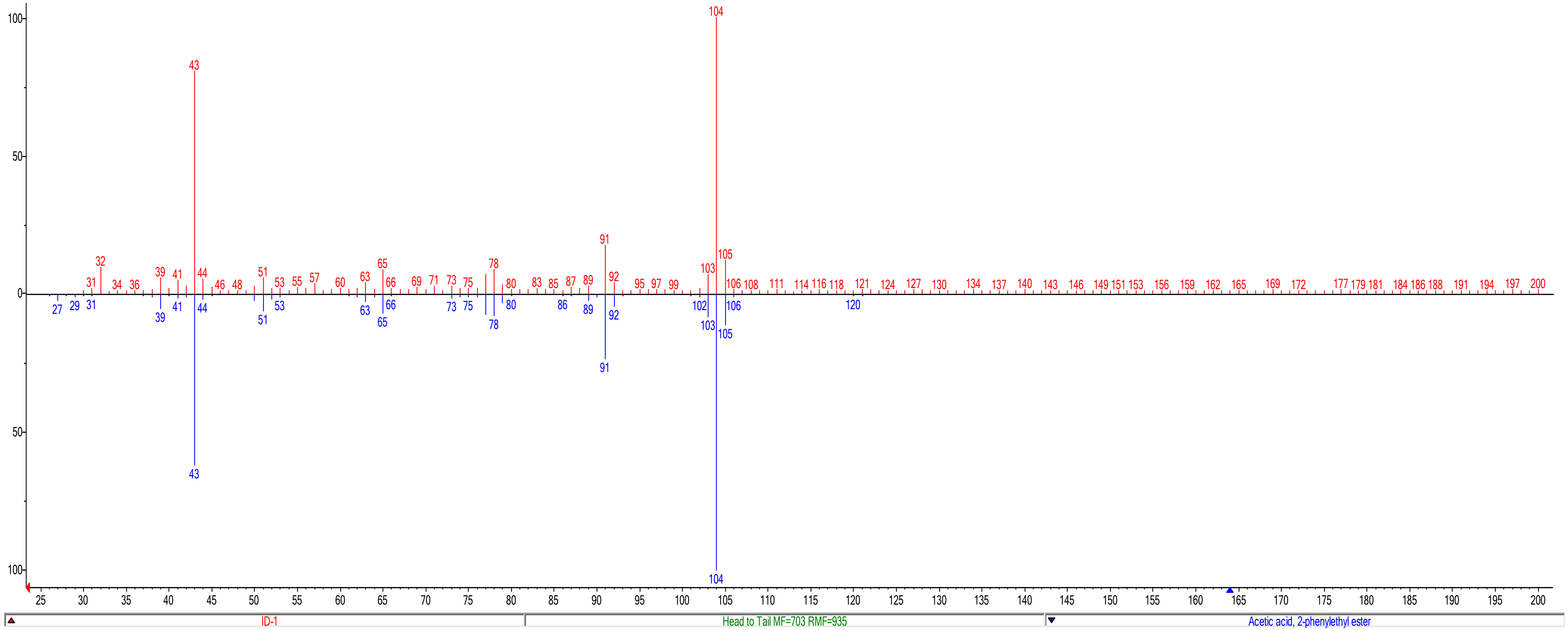

Peak # 124;
RT: 26.249 min
Suggested ID: Nonanoic acid

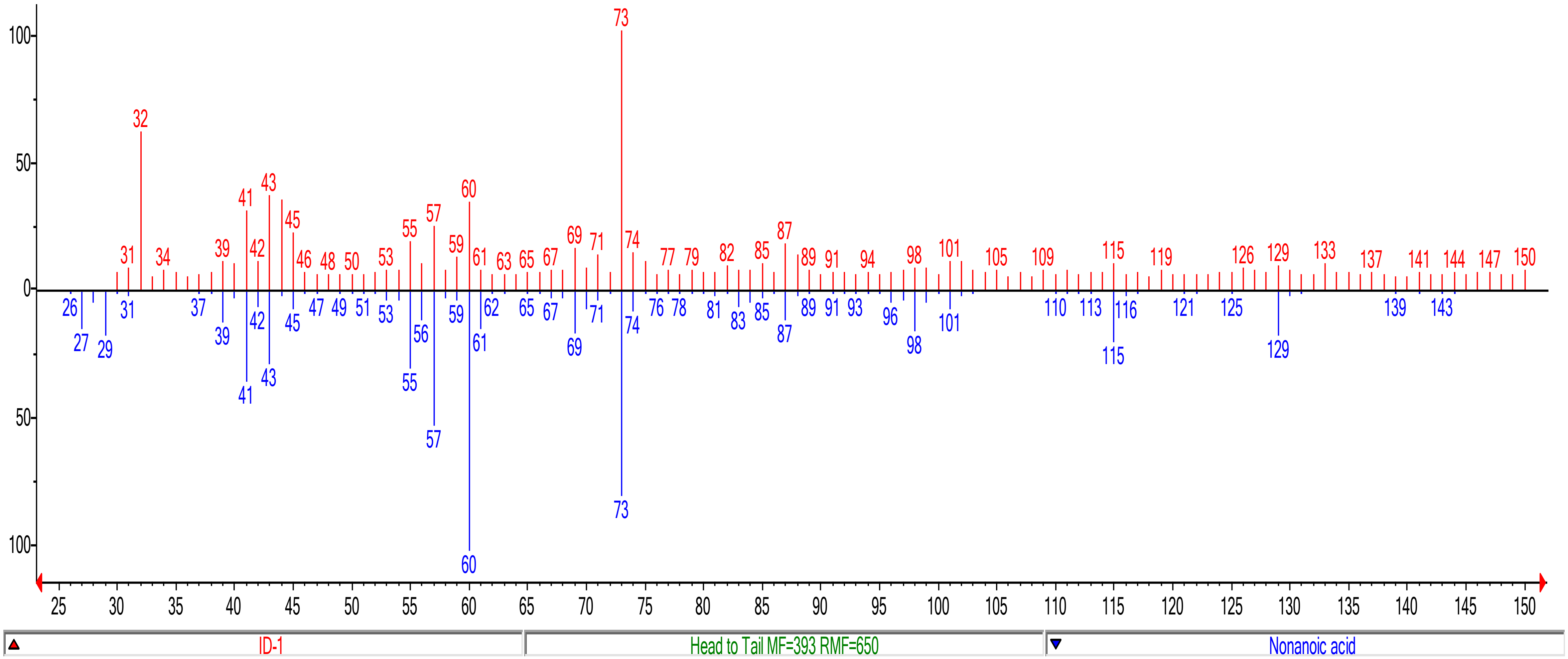

Peak # 129;
RT: 28.952 min
Suggested ID: beta-damascenone

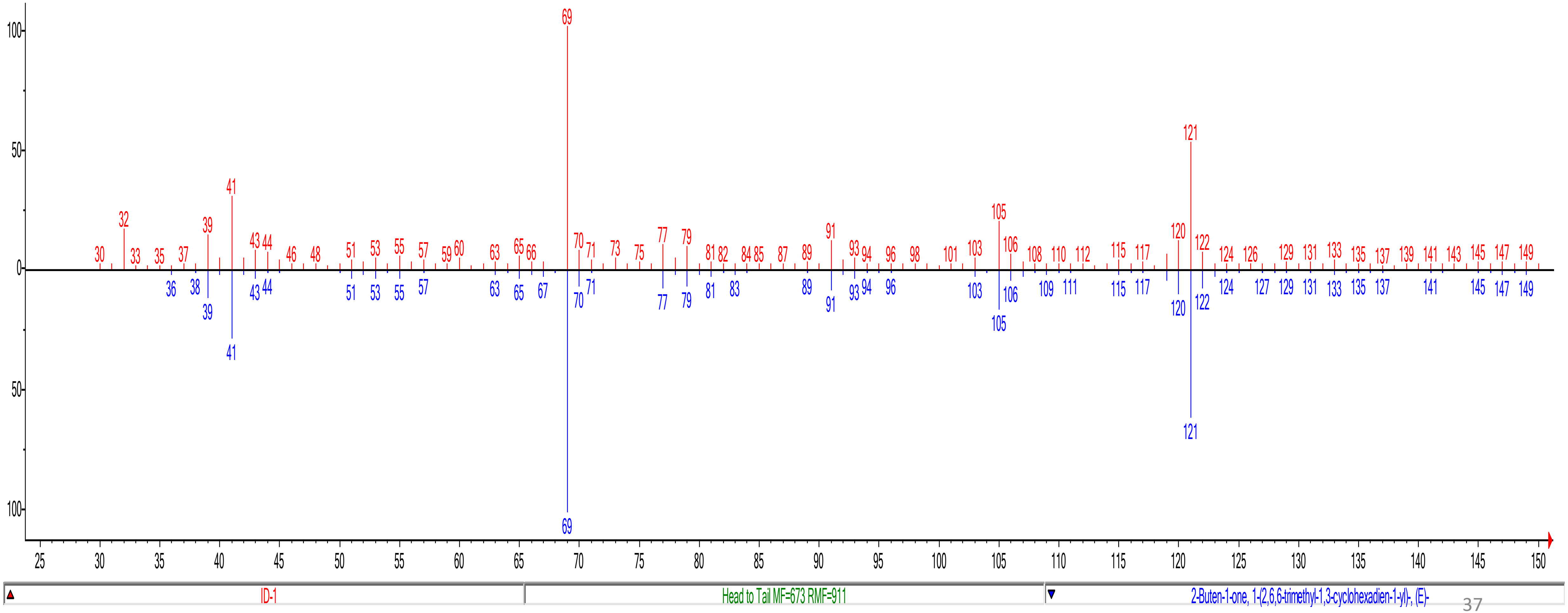

Supplement: Supplementary file 2 [file ECE3-9-8075-s002.pdf]
